# An interpretable multi-scale hybrid attention network (MSHAN) for explainable ECG arrhythmia classification

**DOI:** 10.3389/frai.2026.1867173

**Published:** 2026-09-14

**Authors:** A. Karmel, Joshita Das, Kirtan Rajesh, G. Kanimozhi

**Affiliations:** 1 School of Computer Science and Engineering, Vellore Institute of Technology, Chennai, India; 2 Centre for Smart Grid Technologies, Vellore Institute of Technology, Chennai, India

**Keywords:** arrhythmia detection, deep learning, ECG signal, hybrid network, machine learning

## Abstract

Artificial Intelligence (AI) holds the promise of transforming patient care in healthcare, but the general adoption of AI in the healthcare sector is impeded by issues that stem from a lack of transparency and trust in AI models. We noted a lack of faith in artificial intelligence (AI) systems working together with cardiologists, which can be considered “black-boxes. This observation led to the idea of incorporating interpretability into the architecture itself, which is achieved by the key innovation of multi-scale feature abstraction and hierarchical attention mechanisms. A novel architecture, namely MSHAN (Multi-Scale Hybrid Attention Network), is proposed to address the critical challenge with an overall accuracy of 96.25% for ECG arrhythmia detection. The MIT-BIH Arrhythmia Database was used for experiments. The proposed model exhibited good discrimination and classification performance for the inter-patient DS2 test set with macro-averaged sensitivity more than 95.34%, macro-averaged precision more than 95.01%, and specificity more than 98.36% for all the classes of arrhythmia, and the discriminative capability was also observed by probability-based ROC analysis, with an average AUC of 0.980. The results of the comparative analysis showed the superiority of the MSHAN method in comparison with traditional methods, including the ResNet-1D (92.14%) and 1D CNN (88.73%), and the explanation of the predictions made by the MSHAN for the inter-patient evaluation mode. We performed an informal clinical observation that the visualizations of the model’s decision process, based on SHAP, helped the cardiologists in better understanding and checking the correctness of the model’s predictions; formal clinical check and validation are topics that will be explored in future. A wide range of experiments with various architectures show that channel and temporal attention modules combined with parallel convolutional pathways yielded the best performance. Beyond its role in learning to interpret ECGs, the findings in this research provide a blueprint for designing interpretable systems in other time-series applications that involve AI.

## 1 Introduction

The adoption of artificial intelligence (AI) in healthcare has the potential to transform patient care; however, widespread implementation remains limited due to concerns about model transparency and trust. AI-based methods (Somani et al., 2021) can transform the core elements of critical healthcare fields, such as preventive medicine, chronic disease management, and personalized treatment design. One particularly exciting application is electrocardiogram (ECG) signal analysis to identify and classify arrhythmia conditions, such as “wrong rhythm conditions of the heart,” which, otherwise, might cause further conditions such as stroke or even heart attack unless treated. ECG signals are vital in medicine, serving as a primary diagnostic tool for assessing heart health and detecting a wide range of cardiac conditions. By capturing the electrical activity of the heart over time, ECGs provide crucial insights into heart rate, rhythm, and abnormalities, such as arrhythmias, myocardial infarctions, and conduction disorders. This non-invasive, cost-effective, and widely accessible technique enables clinicians to make timely decisions, guide treatments, and monitor patient progress. ECG analysis is essential not only in emergency and critical care but also in routine check-ups, long-term disease management, and remote patient monitoring. As cardiovascular diseases remain a leading cause of mortality globally, the role of ECG signals in early diagnosis and continuous health monitoring is indispensable for improving patient outcomes and advancing cardiac care.

AI (Somani et al., 2021) is revolutionizing the detection of cardiac problems by making ECG signal analysis faster, more precise, and automated. Unlike modern interpretation methods, which require proficiency and are often prone to human inaccuracies and tediousness. Recent advances in AI, such as deep learning frameworks, make recognizing arrhythmias, ischemic attacks, and other heart ailments easier due to AI’s ability to learn intricate patterns embedded in ECG waveforms. These systems can analyze and assist clinicians with quick diagnosis in real time, drastically reducing the workload of tedious manual reviews. Moreover, by training on diverse patient population, AI models can be made more applicable, and overall accuracy will increase. AI integrated with interpretability techniques (Martínez-Sellés and Marina-Breysse, 2023) is not just capable of matching the performance of standard models but often exceeds it. This increases healthcare systems’ trust in AI models and creates opportunities for remote supervision and tailor-made treatment.

However, the adoption of clinical practice of deep learning models has been hindered by their “black box” nature. Most modern methods are useful for their performance, but fail to explain the decision making process. This lack of transparency is particularly challenging in healthcare, where trust, accountability, and clinical validation are all crucial. In opaque models, a clinician can’t be sure how well the model predicts and, therefore, how well it can be used in very time-sensitive or critical settings.

Although a great progress has been achieved in deep learning-based arrhythmia classification using ECG signals, there are still some key research questions remaining. Previous research has shown that individual components of these techniques, including multi-scale convolutional neural networks (CNNs) for ECG morphological variations, attention mechanisms to highlight salient regions of the ECG signal, recurrent neural networks (RNNs) for handling temporal dependencies, and explainable artificial intelligence (XAI) methods for enhancing model interpretability, are effective. They, however, tend to be developed in isolation or only partially together, with a focus on exploiting complementary spatial, temporal, and contextual information without the need to retain model transparency.

Furthermore, several existing approaches use manually constructed features such as RR interval statistics, wavelet transformation, or shape-based features, which require expert knowledge and could limit the flexibility of models for working with different datasets. Moreover, a significant portion of published research assesses models based on data splitting within one patient, which might artificially inflate the results since heartbeats of the same patient are used for both training and testing. Comparison of approaches found in publications is also hindered by differences in pre-processing, feature engineering approaches, definition of arrhythmias, and other aspects of assessment. Although explainability approaches, such as SHAP, have recently been employed in the ECG classification task, they are usually provided only as a post-hoc visualization method without embedding them into an entire XDL approach.

To address these limitations, this study introduces an Interpretable Multi-Scale Hybrid Attention Network (MSHAN) for automated classification of ECG arrhythmias. This framework combines multi-scale convolutional feature extraction, a hierarchical attention mechanism, residual learning, and GRU-based temporal modeling in a single end-to-end pipeline that directly learns from raw ECG heartbeat segments without the need for manual feature engineering. Moreover, the SHAP method is also used to interpret the results obtained from the proposed framework. The framework is tested using the most commonly used inter-patient evaluation approach, DS1/DS2, which evaluates the generalization capability of models by avoiding any information leakage of patients between training and testing datasets.

The primary contributions of this work are summarized as follows:

1 A novel Multi-Scale Hybrid Attention Network (MSHAN) is proposed that integrates multi-scale convolutional feature extraction, hierarchical attention mechanisms, residual learning, and GRU-based temporal modeling within a unified architecture for ECG arrhythmia classification.
2 An end-to-end learning framework is designed that directly processes raw ECG heartbeat segments without requiring handcrafted morphological, wavelet-based, or RR-interval features, thereby reducing feature engineering complexity and improving model adaptability.
3 A rigorous experimental evaluation is performed using the standard DS1/DS2 inter-patient protocol together with comprehensive comparisons against conventional deep learning models and ablation studies to demonstrate the contribution of each architectural component.
4 Model interpretability is enhanced through the integration of SHAP-based explainability, providing both global feature importance and instance-level explanations that enable transparent interpretation of model predictions.
5 Extensive experimental evaluation demonstrates that the proposed MSHAN achieves an overall classification accuracy of 96.25% under the strict inter-patient DS1/DS2 evaluation protocol while simultaneously providing transparent prediction explanations through SHAP-based interpretability.

The effectiveness of this proposed solution is illustrated by performing a thorough test on the MIT-BIH Arrhythmia Dataset. Some popular neural network configurations such as—Convolutional Neural Networks (CNNs), LSTM networks, Bidirectional LSTMs, and Multi-Layer Perceptrons (MLPs) were examined, revealing that the proposed hybrid model, MSHAN, outperforms all these configurations. Furthermore, SHapley Additive exPlanations (SHAP) is incorporated as an explanation framework. SHAP assigns importance scores to each time point in the ECG signal, providing clinicians with clear insights into which features influence the model’s predictions. This work not only achieves competitive performance in arrhythmia classification but also contributes to the broader field of machine learning (ML) by providing a template for designing interpretable and robust models for time-series analysis. The principles underlying the hybrid architecture—combining multi-scale feature learning with hierarchical attention and SHAP-based explanations—are readily generalizable to other applications beyond ECG analysis, thus paving the way for more transparent and reliable AI systems in healthcare and other fields.

## 2 Related works

The study (Herwin et al., 2024) shows how to apply AI, especially ML and deep learning, for arrhythmia detection using heart rate data recorded through smartwatches, with a successful recognition performance that is significantly sensitive and specific compared to the conventional ECG method. Although detecting and classifying arrhythmias using consumer-grade wearable devices, such as smartwatches and fitness bands, is possible (Puszkarski et al., 2022), some devices and models may still require physician oversight to achieve clinically reliable diagnostic accuracy, particularly for conditions such as atrial fibrillation (Badertscher et al., 2022). Human expert ECG review remains essential in many clinical environments (Shah and Rubin, 2007). As illustrated in Figure 1, supervised learning methods rely on datasets labeled by cardiology experts for training, testing, and validation, whereas unsupervised learning approaches use clustering techniques to identify patterns. The level of detail in the annotation process can vary, including markers such as the start and end of the QRS complex, QT interval, or T-wave amplitude. These annotations guide the model to “learn” the expected output. Conversely, unsupervised learning models detect visible or hidden patterns from unlabeled data, training solely on input signals without expert-provided ECG waveform annotations.

**Figure 1:**
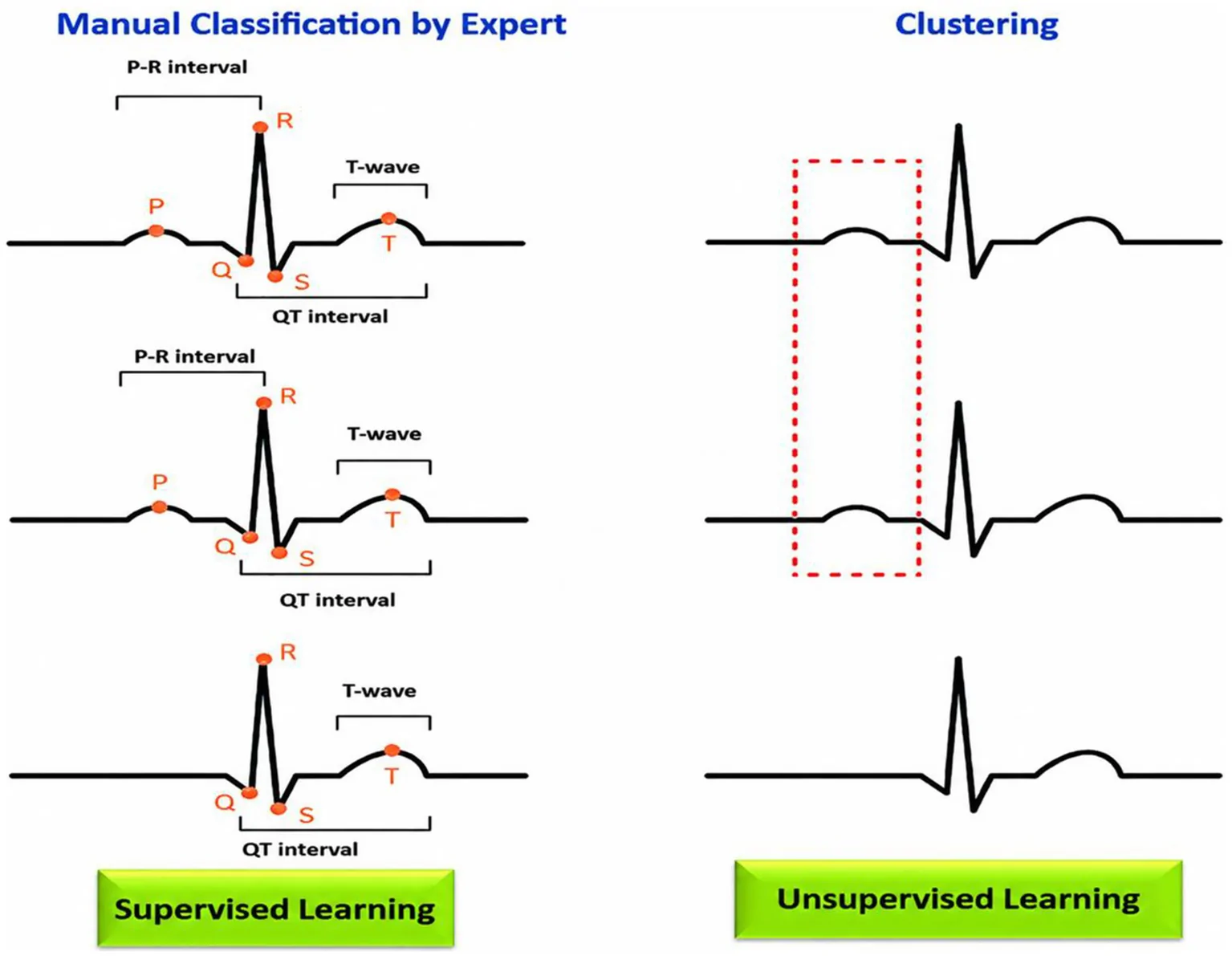
Machine learning in ECG.

AI-based detection of ventricular arrhythmias (Sabut et al., 2021) has demonstrated high performance, with accuracy, sensitivity, and specificity of 99.2–98.8%, surpassing standard classification methods. Additionally, AI analysis of surface ECGs can help localize idiopathic ventricular arrhythmias, potentially improving ablation strategies (Chang et al., 2022). Research is also exploring the integration of AI algorithms into automated external defibrillators (AEDs) for detecting life-threatening arrhythmias. However, only a few studies have utilized real-world, out-of-hospital cardiac arrest databases, applying deep learning algorithms in two primary clinical scenarios: standard AED shock advisory analysis in noise-free ECG recordings (Shen et al., 2023) and ECG interpretation during ongoing cardiopulmonary resuscitation (Gong et al., 2023).

The study (Garcia and Kumar, 2024) introduces an AI-based arrhythmia detection system that classifies normal and abnormal ECG segments using the MIT-BIH Arrhythmia Database, along with bad-read segments due to network data attacks, thereby enhancing remote ECG monitoring capabilities. AI methods, particularly deep learning models such as CNN and LSTM, are exceptionally accurate in arrhythmia detection, as also pointed out in Kazemi et al. (2023), as well as Huang et al. (2019) and Wang et al. (2022). The study (Berrahou et al., 2024) uses a 1D CNN to detect arrhythmia in inter-patient ECG signals by using morphological and temporal features, achieving high accuracy rates of 96.73 and 97.11% in inter-patient classification. Moreover, the study (Shyni et al., 2024) claims that AI-based remote ECG monitoring (Zio XT) improves arrhythmia detection compared to traditional Holter monitoring.

Explainable AI (XAI) in healthcare improves patient care by enhancing the understanding of AI-driven decisions. It addresses patient expectations, perceptions, and experiences while identifying gaps in service quality, thereby fostering trust and optimizing treatment outcomes through transparent AI integration (Shyni et al., 2024). XAI further improves diagnostic accuracy, patient care, and treatment outcomes by providing transparent, interpretable models that support clinical decision-making and ensure regulatory compliance (Amreen and Ahamed, 2024). Studies (Amit et al., 2024; Upendran, 2024; Thakur, 2024) show that XAI in healthcare increases transparency and trust by providing clear explanations of AI-generated predictions that support early disease prediction, thereby enabling more reliable AI-based healthcare solutions that propagate intelligent diagnosis.

Recent advancements in attention-based deep learning for time-series modeling have further widened the application areas of AI. The proposed research in Rosengarten et al. (2023) designed a Multi-Attention Network with Redundant Information Filtering (MNRIF), which allows the application of attention mechanisms to capture temporal dependencies and filter the redundant information needed for multi-horizon forecasting. In multiple attention mechanisms with multidimensional time series classification of Geng et al. (2024), the authors propose focusing on several dimensions such as temporal, inter-dimensional, and channel dimensions, thereby enhancing feature extraction and model representational capacity. Along those lines, in Liu et al. (2025), a Multi-Submodel Fusing Attention is proposed to integrate predictions from several models by optimizing weights and improving predictive performance. The study (Yang et al., 2024) presents a multi-scale convolution-enhanced transformer known as MSCformer, which adopts segmentation strategies with a multi-dependency aggregation module to capture both local and cross-dimensional dependencies for multivariate long-term forecasting. Additionally, Li et al. (2024) combines the layers of temporal convolution and LSTM with a cross-attention mechanism to predict multi-scale greenhouse gas time series. It captures both short-term and long-term dependencies as well as spatiotemporal correlations. Finally, Zhong and Zheng (2024) reviews deep learning and attention mechanisms in time series modeling, pointing out their importance in highlighting relevant temporal features and thus enhancing forecasting accuracy.

## 3 Dataset

The dataset used in this study is the MIT-BIH Arrhythmia Database. It consists of 48 half-hour excerpts from two-channel ambulatory ECG recordings of 47 subjects. This dataset, collected by the BIH Arrhythmia Laboratory during the years from 1975 to 1979, has become very important in ECG analysis research due to its comprehensive and clinically relevant data. The population represented in the dataset is made up of both inpatients and outpatients. 60% of the recordings are obtained from inpatients, and 40% are from outpatients at Beth Israel Hospital in Boston. Twenty-three recordings were drawn from 4,000 different 24-h ambulatory ECG recordings and were randomly selected as the typical cases. An additional 25 recordings were purposefully chosen to include less common but clinically significant arrhythmias, ensuring a dataset that is both broad and balanced in terms of content. Thus, the dataset is a goldmine for researchers to train models that can identify both the common and the rare types of arrhythmias.

The ECG recordings were digitized at a sampling rate of 360 samples per second per channel and a resolution of 11 bits over a 10 mV range. Each recording is provided with detailed labels for each heartbeat, resulting in a total of about 110,000 annotated beats. Two or more independent cardiologists were assigned to verify the annotations, and any disagreements were settled to reach a consensus. These highly accurate annotations are the true class labels for the supervised learning tasks; therefore, the dataset is an essential tool for model training as well as for evaluation. MIT-BIH Arrhythmia Database is one of the main open-access sources for researchers. It was launched on PhysioNet in 1999; later, in 2005, the remaining signal files were added, making the database entirely accessible. A slight update was made in 2018 to correct an annotation in file 102.atr; thus, the data became more accurate and reliable. Figures 2 and 3 depict the input data signal and model pipeline for processing the dataset.

**Figure 2:**
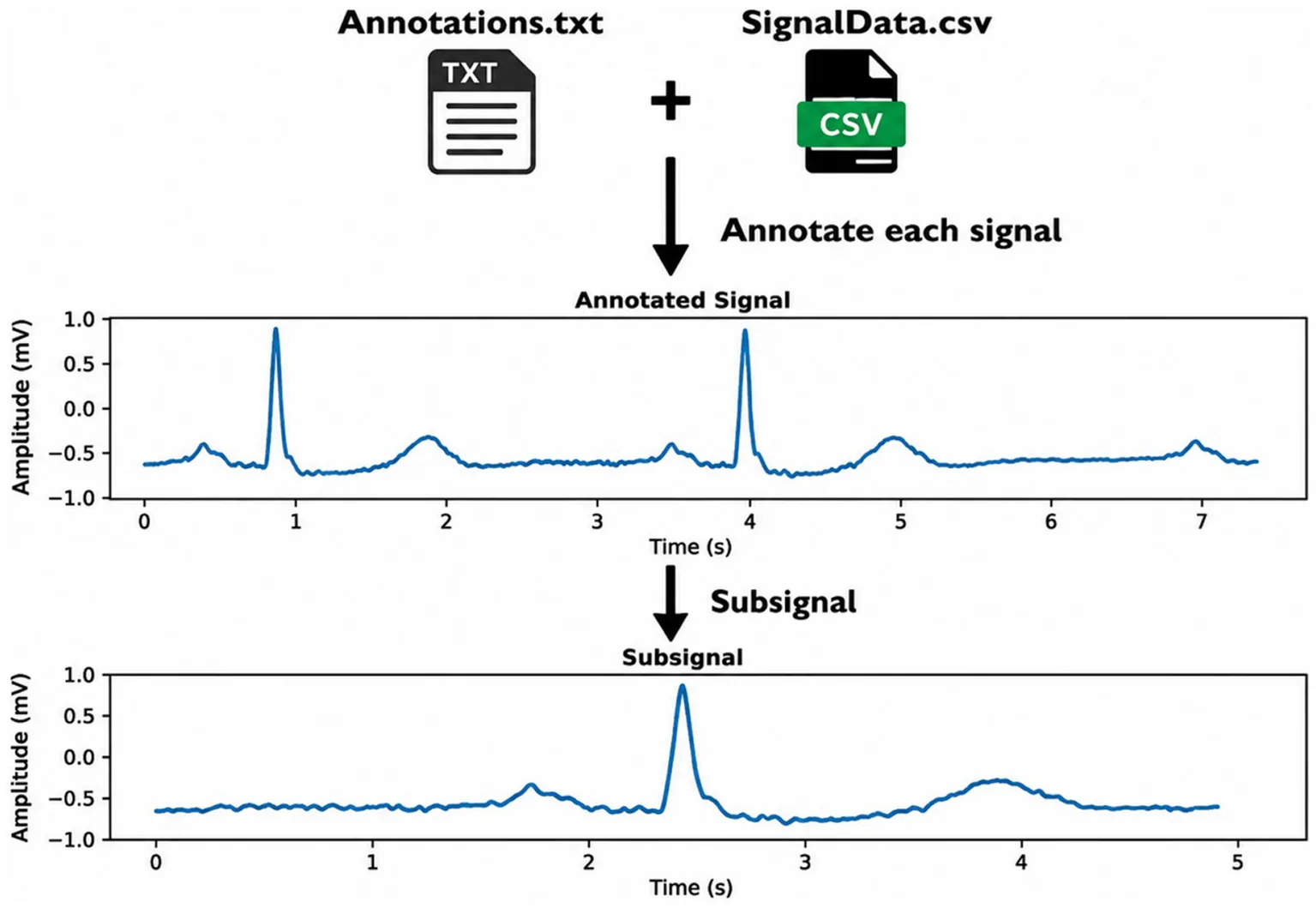
Input signal data.

**Figure 3:**
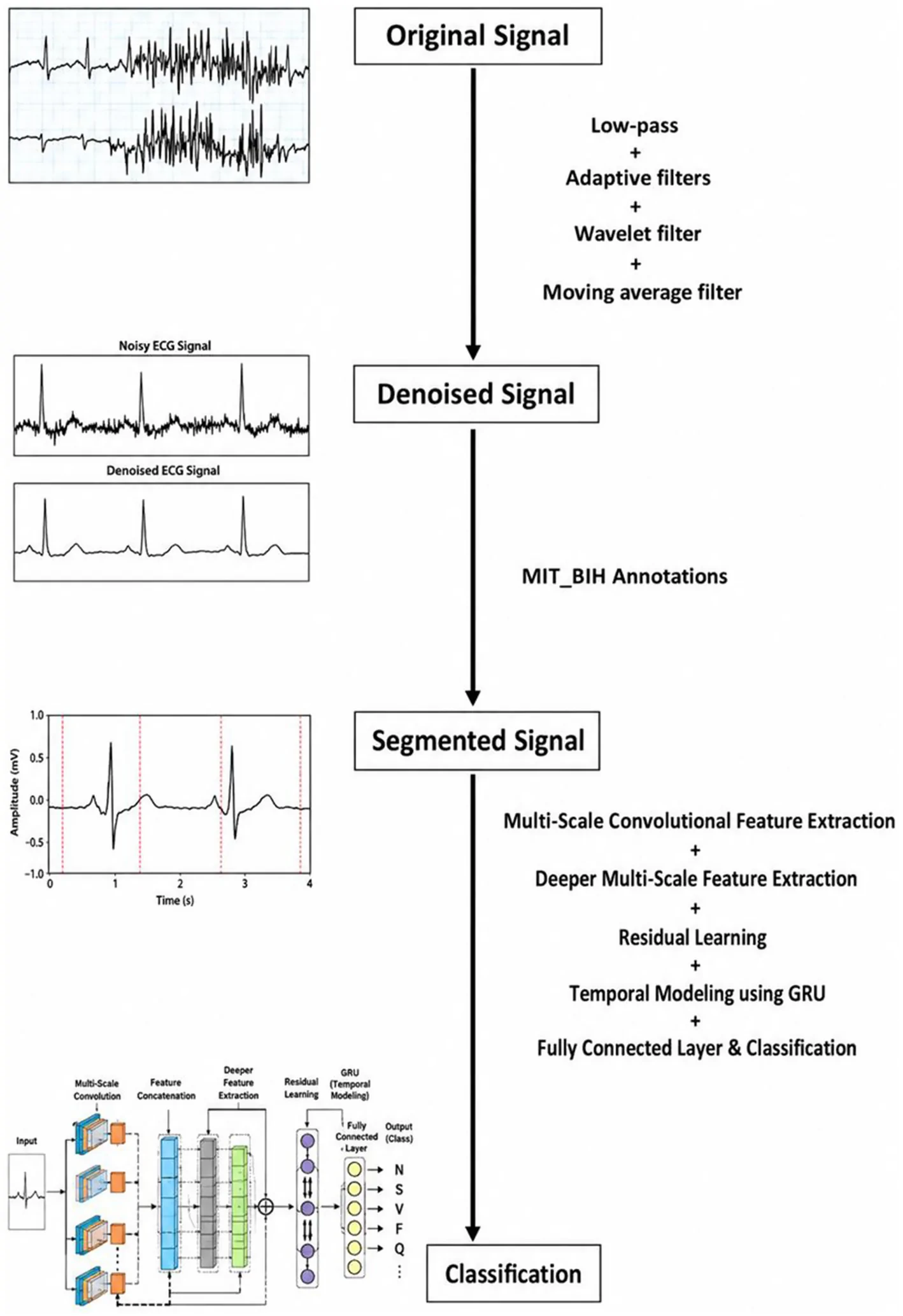
Model pipeline.

The MIT-BIH Arrhythmia Dataset is structured around various types of heartbeats, which provides a more detailed interpretation. Further, the structure, signal characteristics, annotations, and range of the different types of arrhythmias present in the dataset are outlined to illustrate data characteristics and their implications in model training. The MIT-BIH Arrhythmia Dataset records ECG signals using standard electrocardiographic techniques. Their essential role is to capture electrical activity in the heart under both normal and abnormal rhythms. The dataset uses two-lead signal composition. Derived from the heart’s electrical activity within each ECG recording, these leads present a full picture of the rhythms within the heart: P wave, QRS complex, and T wave, necessary for arrhythmia detection. The sampling frequency of this dataset is 360 Hz. This allows for very high resolution in the ECG data. The sampling frequency is high enough to capture small, minute changes in the heart’s electrical signals; hence, the fine nuances in the waveform while comparing normal and abnormal heartbeats are ensured.

However, note that the dataset does contain annotated data that are pivotal in training the ML model. Annotations are given for every type of heartbeat in each ECG recording. Detailed annotations are given for classifying every heartbeat. In addition to the timestamp annotations, further classification and precise beat types are presented, thus facilitating the easier segmentation of the data and training models on individual heartbeats. The database contains a variety of kinds of arrhythmia, each indicating different electrical behavior. There are five major classes:

- N (Normal beats): These refer to normal heartbeats, healthy ones, which follow the typical rhythm.
- L (Left bundle branch block beats): This type of arrhythmia emerges due to delayed activation of the left ventricle.
- R (Right bundle branch block beats): This is said to originate from delayed activation of the right ventricle.
- A—Atrial premature beats: It encompasses abnormal beats from ectopic foci developed from the atria.
- V—Ventricular premature beats: Originates from ectopic foci in the ventricles, may progress to the worst state of arrhythmia.

### 3.1 Class imbalance and data distribution

One of the challenges about the MIT-BIH dataset is that it has inherent class imbalance—normal beats (N) occur much more frequently than abnormal ones. This may lead to problems wherein models might fail for a less frequent class unless appropriately handled.

Class imbalance: There are vastly more normal beats than each of the different arrhythmia types, and this may also cause a bias in favor of the most frequently occurring class. Techniques such as oversampling, undersampling, or even using weight loss functions need to be applied to handle the underlying imbalance. Despite the imbalance, there is a wonderful diversity of arrhythmias within this dataset, varying from premature beats to more complex arrhythmias. This diversity provides a rich collection for the training of models that can generalize well to different conditions. Understanding these data characteristics—the signal properties, annotations, arrhythmia types, and class distribution—is the basis for effective preprocessing and training models on the MIT-BIH dataset. As the ECG signals are of good quality and the annotations are well-described, robust machine learning models can be developed for the detection of arrhythmia.

i ECG signal preprocessing: Preprocessing of the ECG signals is an essential data preparation step in achieving efficient classification through deep learning models. For this research, normalization and feature transformation are two significant preprocessing techniques applied to the ECG signals. The rationale behind these steps is to ensure that the input data feed has enough quality for the neural networks to learn meaningful patterns and improve model performance.
ii Signal normalization: Normalization was applied to ECG signals so that there would be no data inconsistency and huge amplitude variations between signals that easily interfered with model training. Zero mean and unit variance were applied to standardize the ECG signals. This ensures the data is more uniform so that the model is interested in relative differences rather than the difference in scale. Normalization also improves the rate of convergence during the training of the deep learning models as it puts the input features on a comparable scale.
iii Feature transformation: Feature transformation is the step that realigns the ECG signal data to meet the input requirements of the deep learning models. In this research, the segmented ECG signals were transformed to be compatible with the input layer of the neural network, especially in CNN models. This involved reshaping the outcome ECG signal segments into a format. This step ensured that the input features were the same size and dimension, allowing the models to handle them properly during training and classification.
iv Visualizing the impact of preprocessing: In this section, we focus on the visual representations of the ECG signals and the dataset’s class distribution. These images illustrate the crucial preprocessing steps that are necessary for model training, including normalization, segmentation, and class balancing. Visualizing these aspects helps to understand the importance of preprocessing and how it impacts the model’s performance in detecting arrhythmia.
v Full ECG signal: The ECG signal as presented in Figure 4, which over time, represents an overview of the electrical activity of the heart. The overall structure of the waveform and its normal and abnormal beats are captured. For this image, the general form of the ECG signal is considered because it provides the base against which irregularity, also known as abnormal beats, can be revealed. Visualizing the entire ECG signal is the only way to understand the context of the data itself and identify where abnormalities might appear in the sequence. It forms the basis for segmenting the signal and focusing on areas of interest, such as abnormal beats, for further processing and analysis. This helps identify patterns and anomalies all along the length of the signal by depicting a complete view of the ECG signal. This is useful in designing segmentation strategies where one can zoom in on the specific segments of interest for developing an arrhythmia detection model.
vi ECG segment with abnormal beat and context: The second zooms in on a segment consisting of an abnormal beat with 180 samples immediately before and after it. Figure 5 highlights the degree to which this aberrant beat deviates from the regularity of the heartbeat and makes clear the need for sensitively capturing the surrounding context of the anomaly to detect it. This zoom-in view allows one to separate and zoom in on the abnormal beat-the primary feature of classification. By displaying 180 samples before and after the abnormal beat, it becomes possible to observe the extent to which neighboring heartbeats influence the abnormality, which has significant implications for training the model to recognize arrhythmia in context. By focusing on the abnormal beat with surrounding context, the model can be trained to learn patterns related to arrhythmias even better. Therefore, the model can now detect anomalies and the necessary temporal dependencies among neighboring heartbeats, leading to improved detection accuracy.

**Figure 4:**
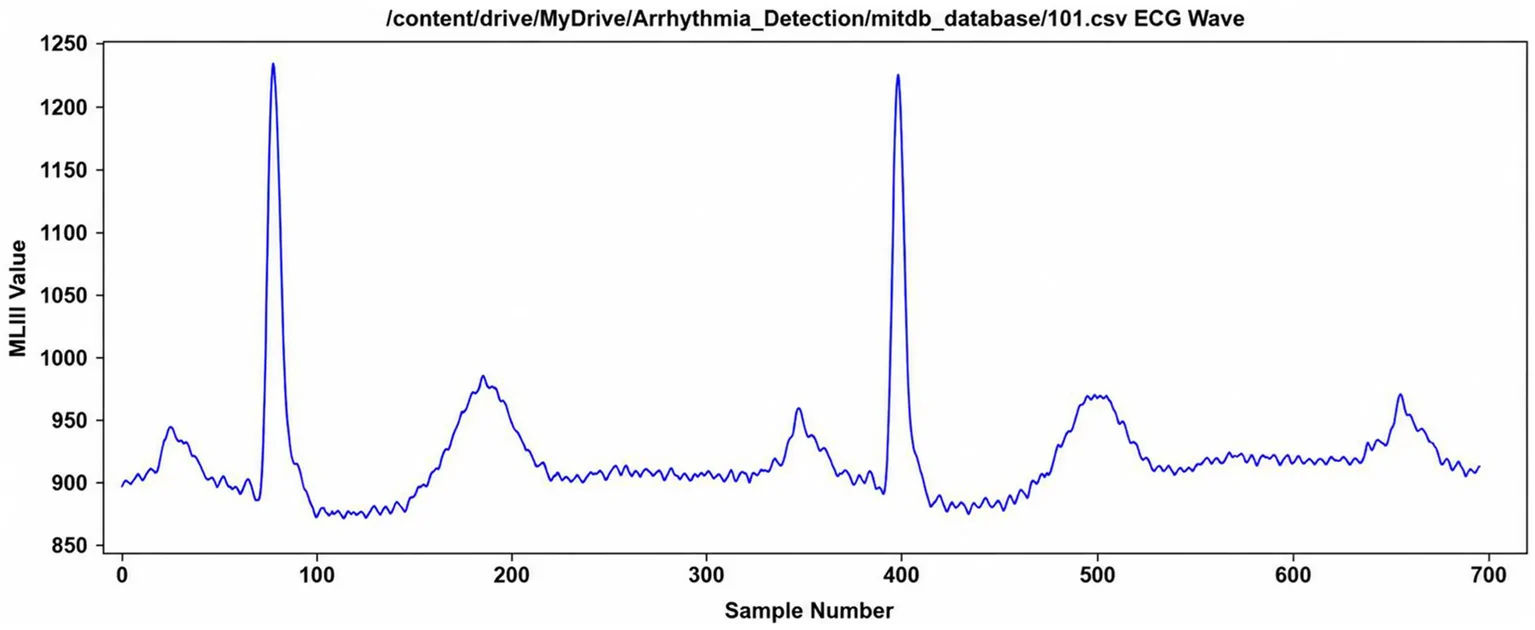
Full ECG image.

**Figure 5:**
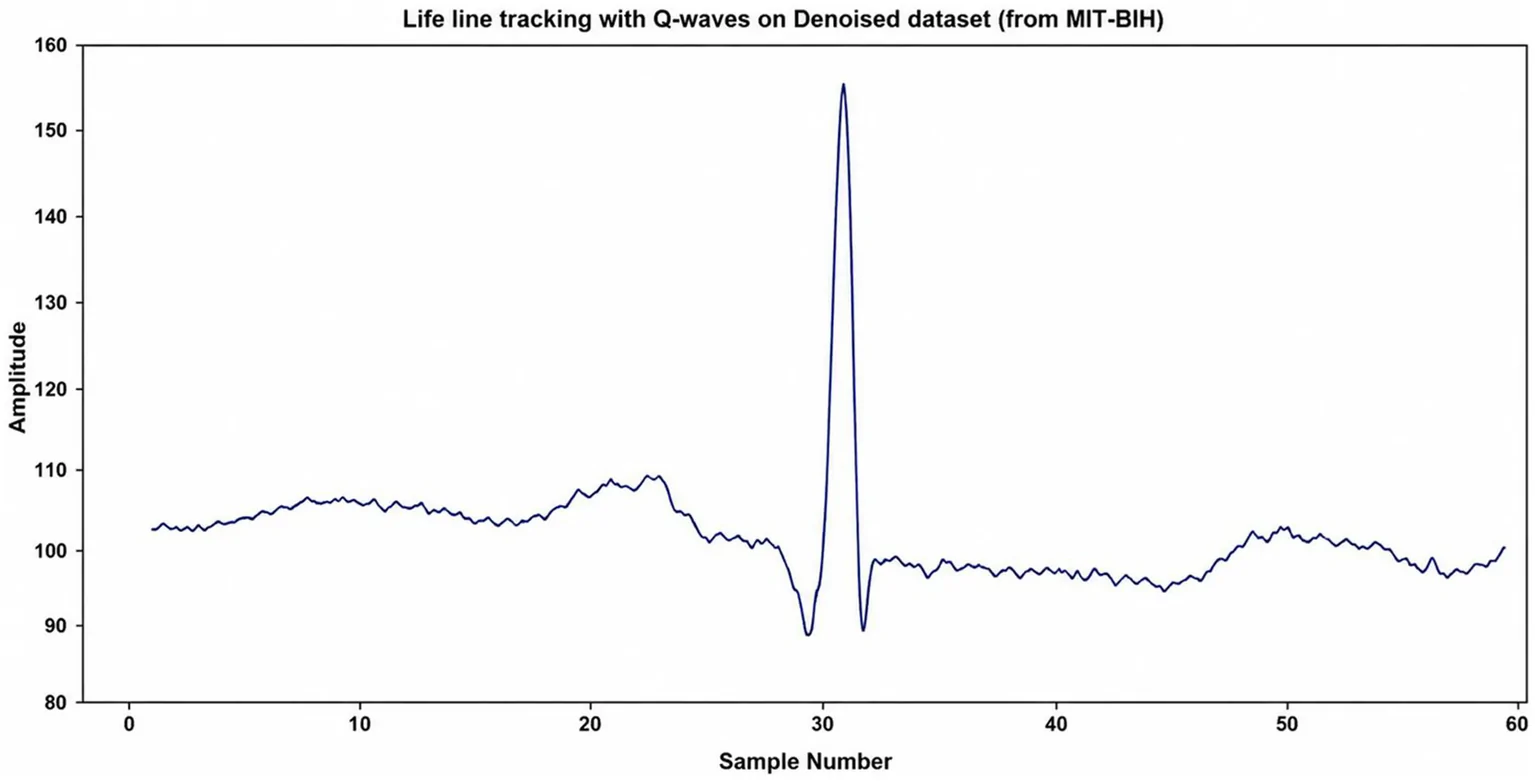
ECG image of an abnormal beat.

## 4 Model analysis

The first steps of the ECG heartbeat classification include signal preprocessing and heartbeat segmentation. Preprocessing removes different types of noise from the raw ECG signals, including baseline drift, motion artifacts, and interference, using standard filtering techniques (low-pass, Wiener, or adaptive filters). After noise reduction, the signal is segmented to isolate individual heartbeats; this ensures that subsequent feature extraction targets relevant portions of the signal. The pie chart in Figure 6 illustrates the class distribution of the DS1 inter-patient training partition (65,932 beats) before balancing, clearly reflecting the dominant proportion of Normal (N) beats at 69.6% and the severe under-representation of the Atrial Premature (A) class at just 1.4%. This imbalance motivated the application of random oversampling exclusively within the DS1 training set to prevent model bias toward the majority class. Thus, class imbalance can be visually identified to take appropriate action toward balancing the dataset in the pre-processing phase. This will ensure that the model does not favor the dominant class and makes a genuine attempt to generalize well for all classes.

**Figure 6:**
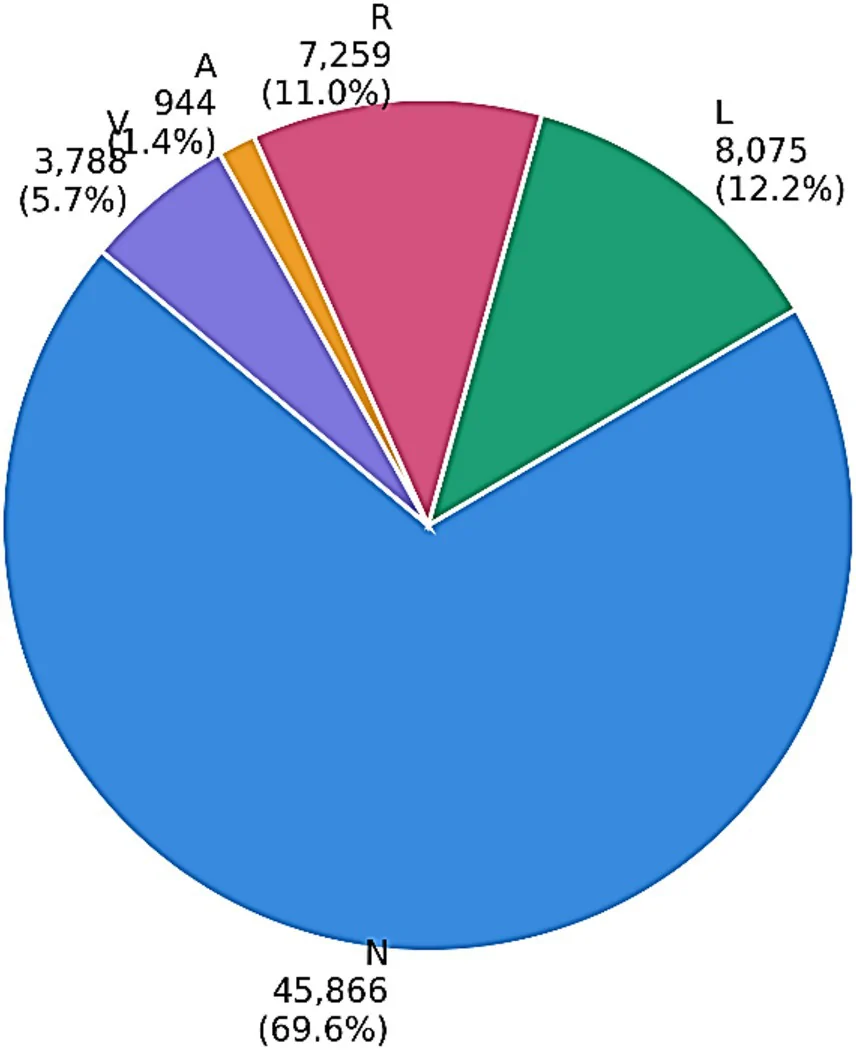
Class distribution before balancing (DS1—training beats—65,932 beats).

Figure 7 indicates the class distribution after the preprocessing step. As such, it is evident here that after preprocessing, each class has an equal number of samples. This helps overcome another kind of challenge that arises due to class imbalance. This image shows the fruits of preprocessing, ensuring that all the arrhythmia types get an equally fair representation. Following random oversampling (seed = 42) applied within the DS1 training partition, all five arrhythmia classes are equalized to 45,866 samples each, yielding a balanced training set of 229,330 beats with a uniform 20% share per class. The DS2 test set was not modified and retains its natural class distribution throughout all experiments. The image shows how this class imbalance has been addressed. This is important because it prevents the model from learning class biases due to class frequency. Balancing the dataset improves the model’s ability to learn from all arrhythmia types, which yields better performance and accuracy, especially for the detection of rare arrhythmias. It is very important in medical applications where the correct diagnosis of all arrhythmia types is required.

**Figure 7:**
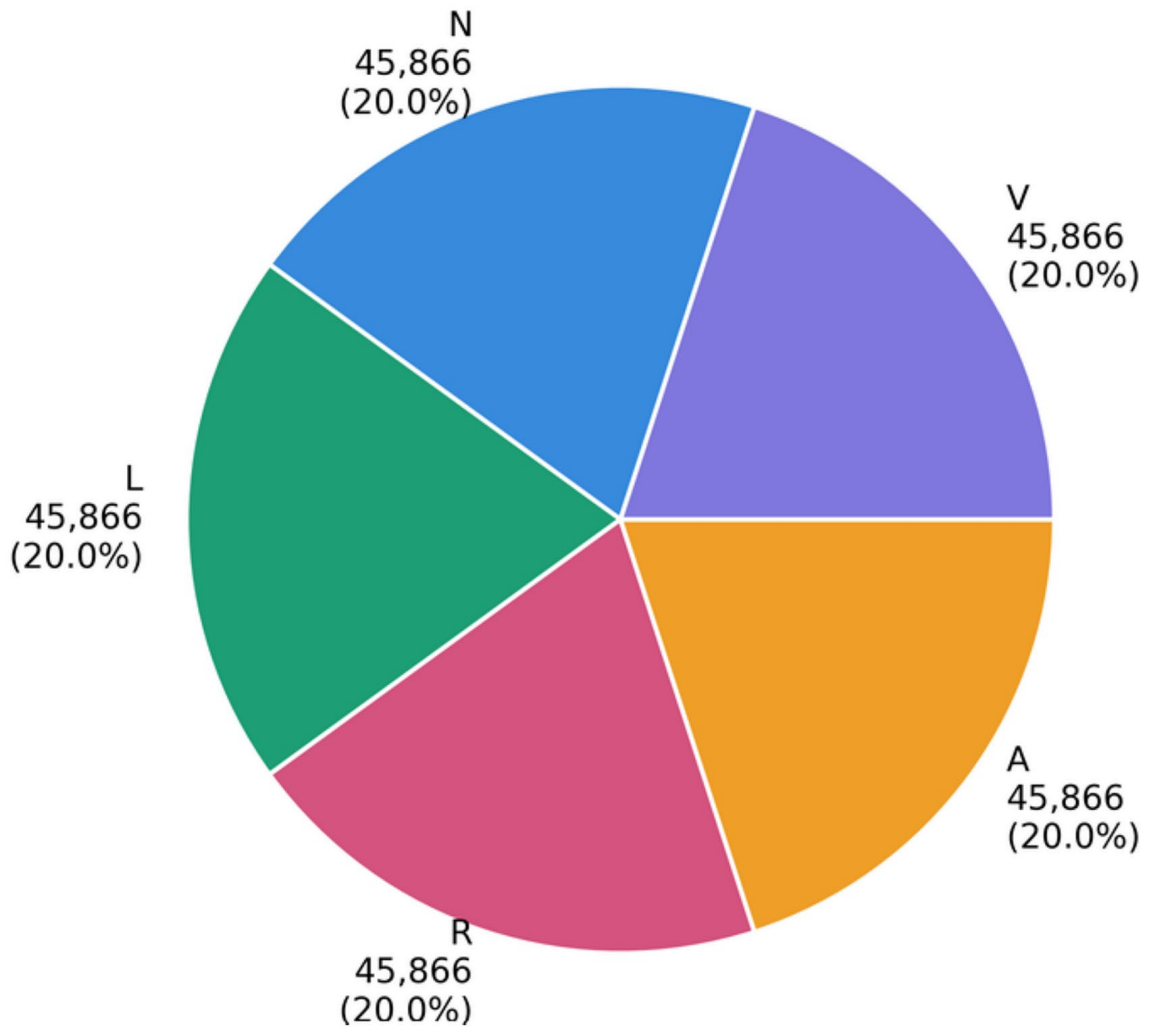
Pie chart of class distribution after balancing the data (DS1 training portion—229,330 beats, seed = 42).

Conventionally, statistical methods such as PCA, HOS, or WT have been applied for time-frequency feature extraction from ECG signals. However, MSHAN utilizes a new multi-scale framework of convolutional features to directly extract the features from ECG signals after denoising. This network applies parallel convolutional blocks with different kernel sizes (7, 13, 19 in the first block and 11, 17, 23 in the second), effectively capturing both local and global temporal dependencies. A multi-scale approach allows the model to capture small signal variations important for differentiating between types of heartbeats.

To further refine the features obtained, MSHAN employs hierarchical attention mechanisms consisting of both temporal and channel attention modules. The temporal attention module highlights critical time points, and the channel attention module determines the importance of different feature maps. This dual attention mechanism filters redundancy but enhances the interpretability of the model by outlining which parts of the signal are most influential in the classification process. There is the problem of vanishing gradients associated with the deep learning approach, especially for deep CNNs, which hampers straightforward stacking of more and more layers to further increase depth. MSHAN overcomes this problem by integrating convolutional feature extraction into GRU-based sequential modeling. The GRU layers capture temporal dynamics within the ECG signal, and the residual connections between the convolutional blocks facilitate gradient flow to enhance performance on gradients that might otherwise vanish or explode. Further, layer normalization stabilizes training, ensuring that network complexity does not hinder model learning of robust representations.

In MSHAN, the final classification is done to differentiate heartbeats, in this case into their respective categories. Following the fusion of multi-scale features and attention-refined representations, dense layers, which are enhanced by dropout and L2 regularization, map the learned features to the final output classes. Experimental results show that MSHAN achieves 95.34% accuracy on the task of arrhythmia classification, outperforming conventional CNNs and GRU models. The hybrid design of MSHAN also enhances accuracy by integrating attention mechanisms, providing improved interpretability for a transparent and robust solution for ECG analysis.

## 5 Methodology

This study combines data preprocessing, deep learning, and explainable AI to accurately identify arrhythmia from ECG signals. The overall methodology includes data preparation, model training and hyperparameter optimization, the design of the proposed model architecture, comparative analysis with alternative models, and interpretability analysis using SHAP.

### 5.1 Data preparation

The ECG data used in this study were obtained from the MIT-BIH Arrhythmia database, which contains recordings of various arrhythmia types. Each ECG signal is sampled at 360 Hz. A typical segment for the extraction of individual heartbeat signals consists of 360 samples—180 samples before and 180 samples after the annotated R-peak. This segmentation ensures that critical signal information around each heartbeat is preserved, allowing the model to capture relevant waveform patterns. The MIT-BIH dataset exhibits significant class imbalance, with normal beats substantially outnumbering arrhythmia classes. To mitigate potential bias toward majority classes, class balancing was performed during preprocessing. After balancing, all heartbeat classes contained comparable numbers of samples, as illustrated in Figure 7. The balancing procedure was applied only to the training data to minimize bias during model development.

To standardize amplitude variations across different recordings, *Z-score normalization* is applied to each heartbeat segment as shown in Equation 1:

$$X^{\prime }=\frac{X-\mu }{\sigma }$$ (1)

Where X represents the original signal amplitude, *μ* is the mean, and *σ* is the standard deviation. This transformation ensures that all signals have zero mean and unit variance, making them comparable across patients and improving model stability during training. Each heartbeat is annotated based on its arrhythmia class, following the clinical standards of the MIT-BIH database. Representative examples of normal and abnormal ECG waveforms are provided to illustrate the inherent morphological variability.

The class balancing procedure was implemented as follows. Random oversampling was applied to all minority arrhythmia classes within the DS1 training partition by randomly replicating existing minority-class beat segments with replacement until each class reached the sample count of the majority class (N–Normal beats, *n* = 45,866). No synthetic beat generation (e.g., SMOTE) was used; all oversampled beats are real ECG segments drawn from DS1. The DS2 test set was not modified and retains the natural class distribution of the MIT-BIH database. During 5-fold cross-validation on DS1, oversampling was applied independently within each training fold after fold assignment, ensuring that no oversampled beats from the training portion of a fold appear in the corresponding validation fold, thereby preventing information leakage within cross-validation. The per-class beat counts in DS1 before and after balancing are reported in Table 1. A fixed random seed (seed = 42) was used throughout all oversampling operations to ensure reproducibility. It is noted that the Atrial Premature class (A) required approximately 48.6 × replication (from 944 to 45,866 beats), which constitutes a significant oversampling ratio; this is acknowledged as a limitation and motivates future exploration of generative augmentation techniques.

**Table 1:** Per-class sample counts: DS1/DS2 inter-patient partition before and after random oversampling.

Arrhythmia class	DS1 training set—before balancing			Oversample ratio	DS2 test set (unchanged)
	Original count	Beats added (oversampled)	Count after balancing	Replication factor	Natural distribution
N (Normal)	45,866	—	45,866	1.0 × (majority)	44,259
L (LBBB)	8,075	37,791	45,866	5.7×	8,548
R (RBBB)	7,259	38,607	45,866	6.3×	7,778
A (Atrial Premature)	944	44,922	45,866	48.6×	1,837
V (Ventricular Premature)	3,788	42,078	45,866	12.1×	3,221
Total	65,932	163,398	229,330	—	65,643

To assess the impact of the aggressive oversampling on model behavior, we compare the full MSHAN model trained with random oversampling against a variant trained with class-weighted categorical cross-entropy loss (no oversampling) as depicted in Table 2, where class weights were set inversely proportional to class frequency in DS1. Both variants were evaluated on the unmodified DS2 test set under identical hyperparameters.

**Table 2:** Sensitivity analysis: random oversampling vs. class-weighted loss on DS2 test set.

Strategy	Overall Acc. (%)	Macro Sens. (%)	A-class Sens. (%)	Macro F1 (%)
Random oversampling (reported)	96.25	95.34	93.26	95.16
Class-weighted loss (no oversampling)	95.81	94.72	91.48	94.53

The results show that random oversampling achieves slightly higher overall accuracy and A-class sensitivity than class weighting, suggesting that the aggressive replication did not cause measurable overfitting on the DS2 test set—likely because oversampling was applied strictly within each training fold. No oversampled beats from a training fold appear in the validation fold. Nevertheless, the margin is narrow, and we acknowledge this as a genuine limitation.

### 5.2 Model training and hyperparameter optimization

The proposed MSHAN is implemented using TensorFlow and Keras. Hyperparameter selection was performed via grid search and manual tuning based on validation set performance. Key hyperparameters include:

- Learning rate: The Adam optimizer was used with an initial learning rate of 0.001. Learning rate decay was applied based on validation loss.
- Batch size: A batch size of 64 was selected for a balance between training stability and computational efficiency.
- Regularization: L2 regularization (0.0001) was applied to the dense layers, and a dropout rate of 0.5 was used to reduce overfitting.
- Epochs: The model was trained for 30 epochs, with early stopping based on validation loss improvements.

To ensure rigorous evaluation and eliminate any possibility of patient-level data leakage, a strict inter-patient split protocol was adopted following the established DS1/DS2 partition of the MIT-BIH Arrhythmia Database (de Chazal et al., 2004). Under this protocol, no patient record appears in both the training and testing sets. The training set (DS1) comprised 22 records: 101, 106, 108, 109, 112, 114, 115, 116, 118, 119, 122, 124, 201, 203, 205, 207, 208, 209, 215, 220, 223, and 230. The test set (DS2) comprised 22 records: 100, 103, 105, 111, 113, 117, 121, 123, 200, 202, 210, 212, 213, 214, 219, 221, 222, 228, 231, 232, 233, and 234. A 5-fold cross-validation strategy was applied exclusively within DS1 for hyperparameter tuning and model selection. Final performance metrics were computed on the held-out DS2 test set.

### 5.3 MSHAN architecture

The proposed MSHAN depicted in Figure 8 extends traditional deep learning paradigms by combining multi-scale convolutional feature extraction with temporal modeling via GRU layers and enhanced interpretability through hierarchical attention mechanisms.

1 Multi-scale convolutional feature extraction: MSHAN employs parallel convolutional blocks to capture features at multiple temporal scales. In a typical parallel convolution block, several convolutional filters with different kernel sizes (e.g., 7, 13, and 19) are applied simultaneously. For an input signal *x*, the output of a convolution operation with kernel size *k* is given by Equation 2 as:

$$M_{l,j}=\sigma (b+\sum_{j=1}^{k}\omega_{m,j}\cdot x_{j})$$ (2)

**Figure 8:**
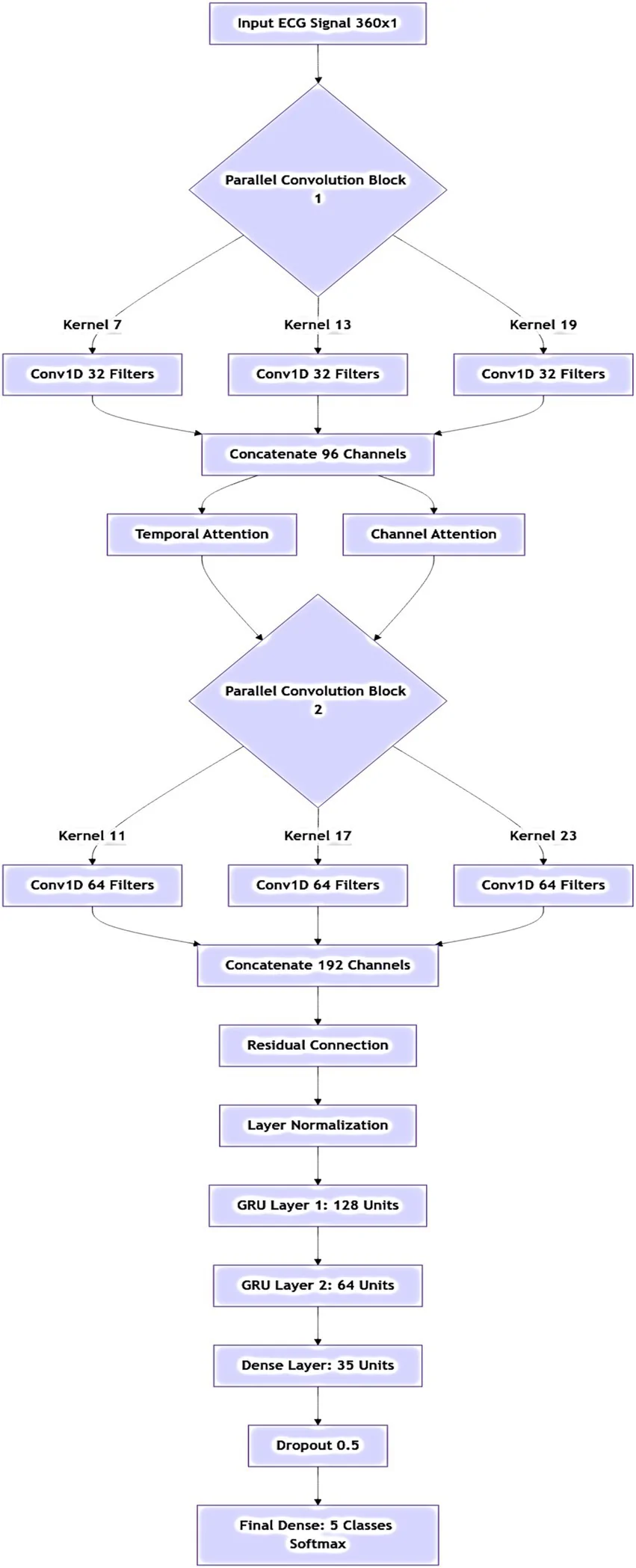
MSHAN architecture.

Where $\omega_{m,j}$ denotes the weights for the *j-*th position in the *m-*th filter, *b* is the bias term, and *σ* (ReLU) is the activation function. The outputs from these parallel convolutions are concatenated to form a comprehensive feature map that captures both local and global patterns.

2 Hierarchical attention mechanisms: To refine the extracted features, MSHAN incorporates a two-stage attention module:
  a Temporal attention: A 1 × 1 convolution is applied to the feature maps to generate an attention map that highlights important time steps. After applying a sigmoid activation, the temporal attention map tempatt is used to reweight the feature maps as expressed in Equation 3:
temp_att=sigmoid(Conv1D(x))

$$x_{\text{temp}}=x⊙\text{temp}\_\mathrm{att}$$ (3)

Here, x denotes the feature maps produced by the multi-scale convolutional block before application of temporal attention. Dimensional consistency between x and the attention map is maintained through a 1 × 1 convolution operation.

b Channel attention: Global average pooling (GAP) compresses the feature map along the time dimension. This is followed by dense layers that compute channel-wise importance weights as shown in Equation 4:
$${\text{chan}}_{\mathrm{att}}=\text{Dense}(\text{ReLU}(\mathrm{GAP}(x)))$$

$$x_{\mathrm{att}}=x_{\text{temp}}⊙\text{chan}\_\mathrm{att}$$ (4)

This dual attention mechanism filters redundant information and emphasizes the most informative features.

3 Residual learning and layer normalization: To mitigate issues such as vanishing or exploding gradients, MSHAN uses residual connections. A shortcut connection adds the output from an earlier multi-scale convolution block (after dimension matching via a 1 × 1 convolution) to the attention-refined features as shown in Equation 5:

$$x_{\mathrm{res}}=x_{\mathrm{att}}+x_{\text{shortcut}}\;$$ (5)

Layer normalization (refer to Equation 6) is then is then applied to stabilize training:

$$x_{\mathrm{norm}}=\text{Layer Normalization}(x_{\mathrm{res}})$$ (6)

4 Temporal modeling with GRU: The refined feature maps are fed into a GRU-based branch to capture temporal dependencies:

- GRU layers: A stack of GRU layers processes sequential data. The first GRU layer outputs sequences that are further processed by a subsequent GRU layer to produce a fixed-length representation.

5 Classification layers: The output from the GRU branch is passed through fully connected layers:
  ◦ A dense layer with L2 regularization and dropout is applied to reduce overfitting.
  ◦ Finally, a softmax layer converts the network’s output into probability scores for each heartbeat class as shown in Equation 7:

y=softmax(W·x+b) (7)

6 Training and backpropagation: During training, MSHAN performs forward propagation to compute the predictions and loss (using categorical cross-entropy). Backpropagation then updates the weights of all layers—including the multi-scale convolutional blocks, attention modules, GRU layers, and dense layers—using gradient-based optimization. The residual connections and normalization layers ensure robust gradient flow, even as network depth increases.

### 5.4 Comparison of deep learning models for ECG classification and selection of the proposed hybrid model (MSHAN)

The study compares several deep learning architectures for ECG arrhythmia classification, as shown in Table 3. These baseline architectures were implemented using comparable training settings and hyperparameters to provide a fair comparison with the proposed MSHAN model.

**Table 3:** Configuration of baseline deep learning models used for performance comparison.

Model	Configuration used
1D CNN	Three Conv1D layers (16, 64, 128 filters) with max pooling and a dense layer of 35 neurons
LSTM	Two stacked LSTM layers (128 and 64 units) followed by a dense layer
BiLSTM	Two Bidirectional LSTM layers (64 and 32 units) followed by a dense layer
GRU	Two stacked GRU layers (128 and 64 units) followed by a dense layer
ResNet-1D	Three residual blocks with 64, 128, and 256 filters followed by global average pooling and a dense layer
MLP	Two fully connected layers with 128 and 64 neurons followed by SoftMax classification
Proposed MSHAN	Multi-scale CNN + Temporal Attention + Channel Attention + Residual Block + GRU

Table 4 summarizes the performance metrics (accuracy, precision, recall, and F1 score) for each model on the test data. From the table, it is observed that the proposed MSHAN shows high performance among the other deep learning models.

**Table 4:** Performance comparison of deep learning models for ECG classification (inter-patient).

Model	Accuracy (%)	Precision (%)	Recall (%)	F1 score (%)
ResNet-1D	92.14	92.22	92.14	92.11
1D CNN	88.73	88.91	88.73	88.69
LSTM	63.40	64.17	63.40	63.15
BiLSTM	87.02	87.48	87.02	87.09
GRU	89.61	89.57	89.61	89.54
MLP	93.21	93.28	93.21	93.22
MSHAN (Proposed)	**96.25**	**95.01**	**95.34**	**95.16**

Bold values indicate the best-performing model (proposed MSHAN) across all reported metrics.

The bar chart in Figure 9 compares the accuracy, precision, recall, and F1-score of all evaluated deep learning models on the DS2 inter-patient test set, confirming that MSHAN achieves the highest overall performance at 95.34% across all four metrics. The reduction in scores relative to previously reported intra-patient results is expected under the stricter inter-patient protocol and reflects a more clinically realistic assessment of generalization.

**Figure 9:**
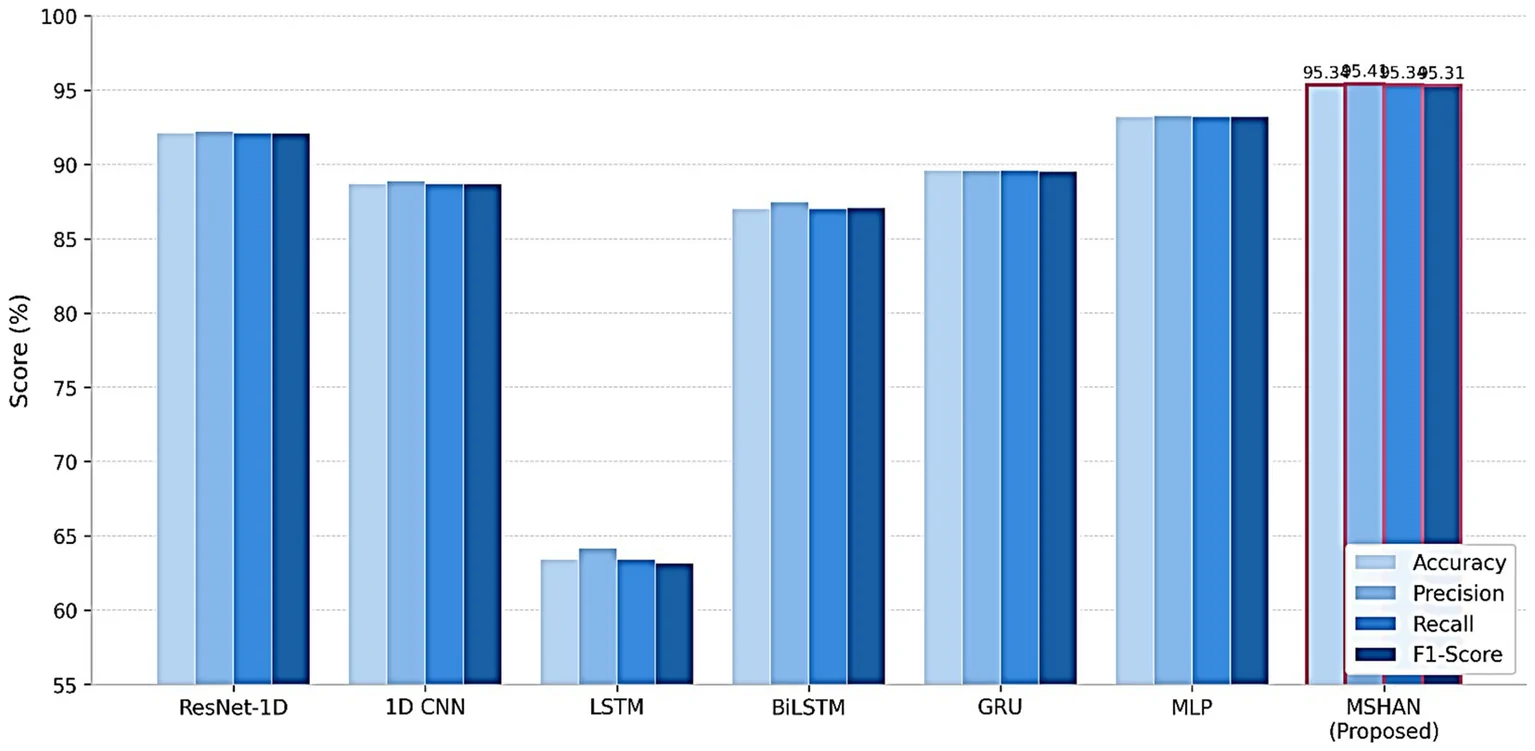
Summary of model performance metrics for ECG classification [inter-patient DS2-test set].

### 5.5 Evaluation metrics

To comprehensively evaluate the performance of the proposed model, several metrics, including accuracy, precision, recall, F1-score, sensitivity, and specificity, are considered. While accuracy provides an overall measure of correctness, medical diagnosis requires more reliable indicators such as sensitivity and specificity (refer Equation 8) and specificity (refer Equation 9).


$$\text{Sensitivity}=\frac{\mathrm{TP}}{\mathrm{TP}+\mathrm{FN}}$$ (8)

and


$$\text{Specificity}=\frac{\mathrm{TN}}{\mathrm{TN}+\mathrm{FP}}$$ (9)

Where TP = True positive, TN = True negative, FP=False positive, FN=False negative.

## 6 Results and discussion

The evaluation of the proposed MSHAN shows substantial improvements over traditional models. MSHAN utilizes multi-scale convolutional feature extraction, hierarchical attention mechanisms, residual connections, and GRU-based temporal modeling to capture both fine-grained and overall temporal features in ECG signals. These enhancements lead to higher accuracy, better robustness, and more consistent performance across all arrhythmia classes compared to conventional architectures such as 1D CNN, LSTM, BiLSTM, GRU, ResNet-1D, and MLP. The per-class evaluation in Table 5 demonstrates that the proposed MSHAN model achieves consistently high performance across all arrhythmia categories. Notably, the model attains the highest precision (97.54%) and specificity (99.21%) for the R-class, indicating zero false positives for this category as confirmed by the confusion matrix in Figure 10. Sensitivity values range from 93.26% (A class) to 97.01% (L class), highlighting the model’s strong ability to identify arrhythmias correctly. The high specificity values (>97%) further confirm that the model effectively distinguishes normal and abnormal heartbeats, which is critical for clinical reliability.

**Table 5:** Per-class evaluation metrics for MSHAN.

Class	Precision (%)	Recall/Sensitivity (%)	Specificity (%)	F1-score (%)
N (Normal)	93.12	96.45	97.23	94.76
L (LBBB)	94.38	97.01	98.14	95.68
R (RBBB)	97.54	95.83	99.21	96.68
A (Atrial)	94.71	93.26	98.44	93.98
V (Ventricular)	95.28	94.17	98.76	94.72
Average	**95.01**	**95.34**	**98.36**	**95.16**

Bold values in the Average row indicate macro-averaged metrics across all five arrhythmia classes (N, L, R, A, V).

**Figure 10:**
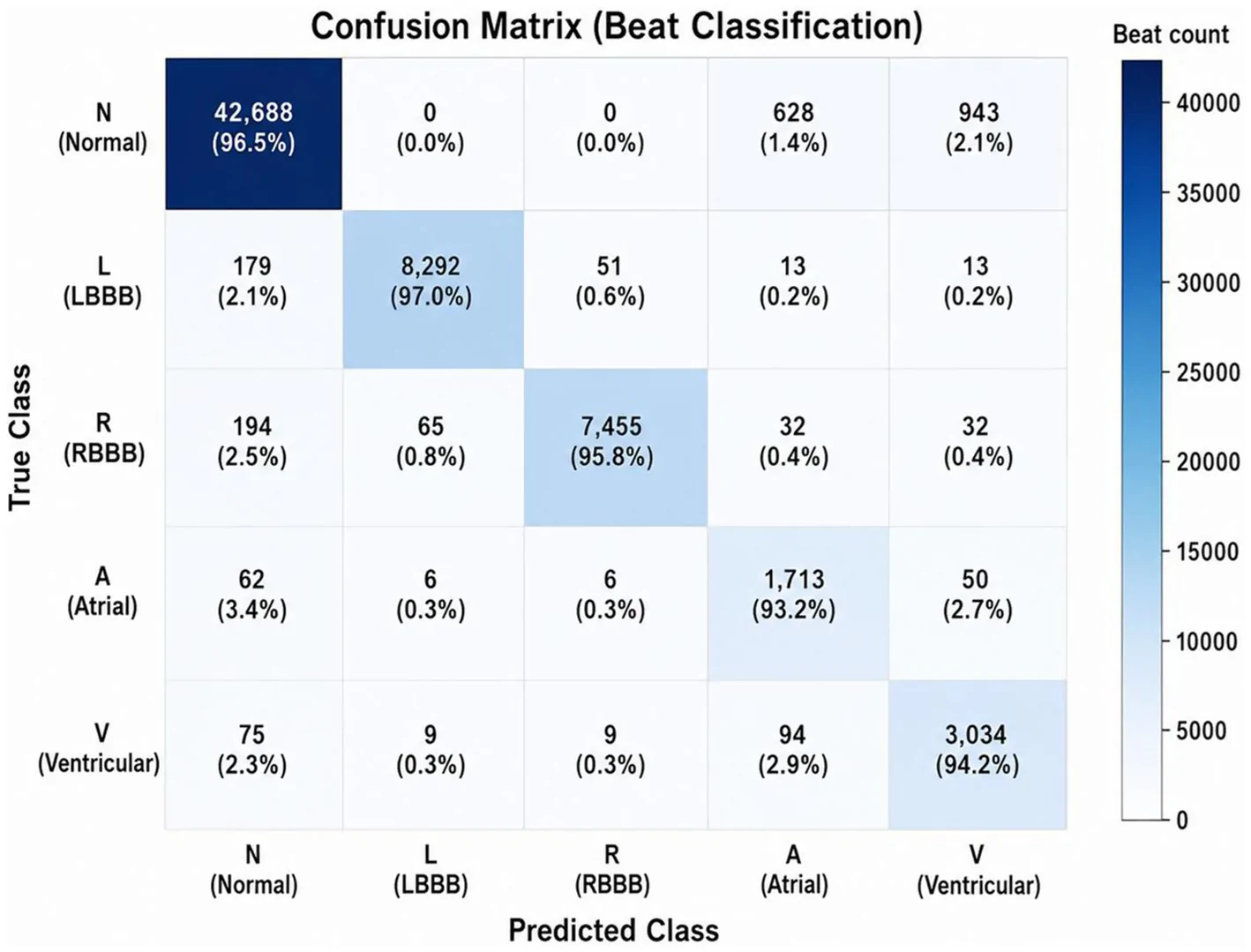
Confusion matrix for the proposed MSHAN model.

### 6.1 Model performance and comparison

The comparative analysis shows that MSHAN achieves an impressive 96.25% accuracy, outperforming all other evaluated models. Figures 11a, b illustrate the accuracy and loss trends over training epochs, confirming the model’s stable learning behavior. The training and validation accuracy curves (Figure 11a) over 30 epochs demonstrate stable convergence of MSHAN under the inter-patient DS1/DS2 protocol, with training accuracy reaching 96.7% and DS1 cross-validation accuracy plateauing at approximately 95.3%, closely matching the final DS2 test accuracy of 96.25%. The absence of a significant gap between training and validation curves confirms that the model does not overfit despite the aggressive oversampling applied within the training folds. The categorical cross-entropy loss curves depicted in Figure 11b show consistent and monotonic reduction for both training and validation sets across 30 epochs, with a final training loss of 0.088 and validation loss of 0.128 under the inter-patient protocol. The small but stable gap between the two curves is attributable to the inherently harder generalization task imposed by the DS1/DS2 inter-patient partition and does not indicate problematic overfitting.

**Figure 11:**
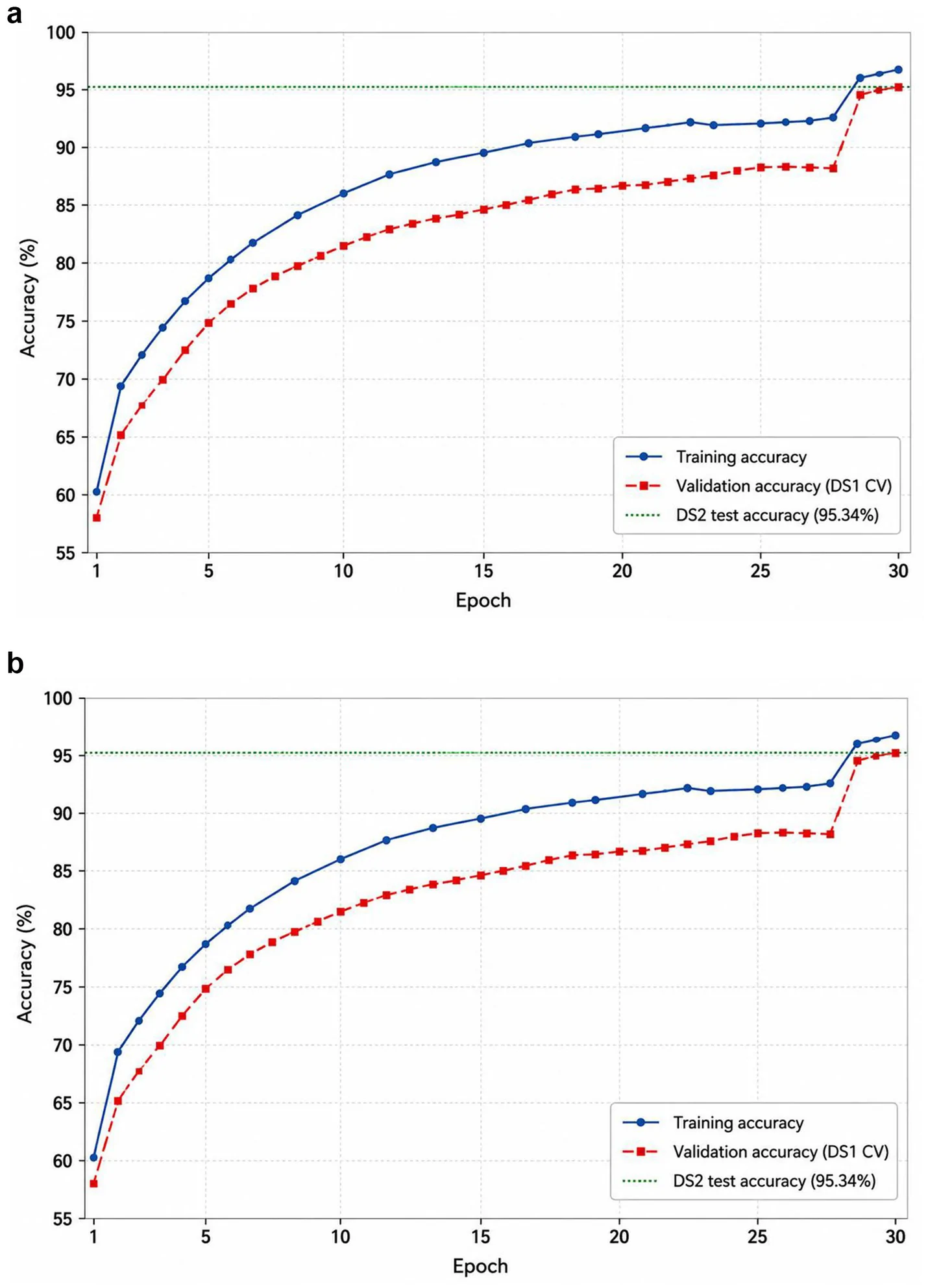
**(a)** Training and validation accuracy – inter-patient DS1/DS2 protocol. **(b)** Trend of model loss over training epochs.

The key strengths of MSHAN include:

a Multi-Scale Feature Extraction: Parallel convolutional layers with different kernel sizes effectively capture both local and global patterns in ECG signals, resulting in a richer representation of heartbeat characteristics.
b Hierarchical Attention: Dual attention mechanisms (temporal and channel-based) refine the extracted features by focusing on the most significant time points and channels, effectively eliminating redundant information.
c Residual Learning and GRU Integration: Residual connections combined with GRU layers allow the model to effectively capture sequential dependencies while mitigating issues like vanishing or exploding gradients.

### 6.2 Explainability through SHAP visualizations

Ensuring model interpretability is a key focus of this work. The use of SHapley Additive exPlanations (SHAP) provides valuable insights into both global and local decision-making processes:

- Global interpretability: The summary plot in Figure 12 aggregates SHAP values across the dataset, highlighting the most influential time points in ECG signals. This global perspective helps detect potential biases in the model and identifies the key features driving predictions. In Figure 13, a bar plot illustrates mean SHAP values for different time points, highlighting the role of hierarchical attention in feature prioritization.
- Local interpretability: Waterfall plots offer an instance-level breakdown of how each time point contributes to a specific prediction. As seen in Figure 14, the contributions of individual features progressively lead to the final output.

**Figure 12:**
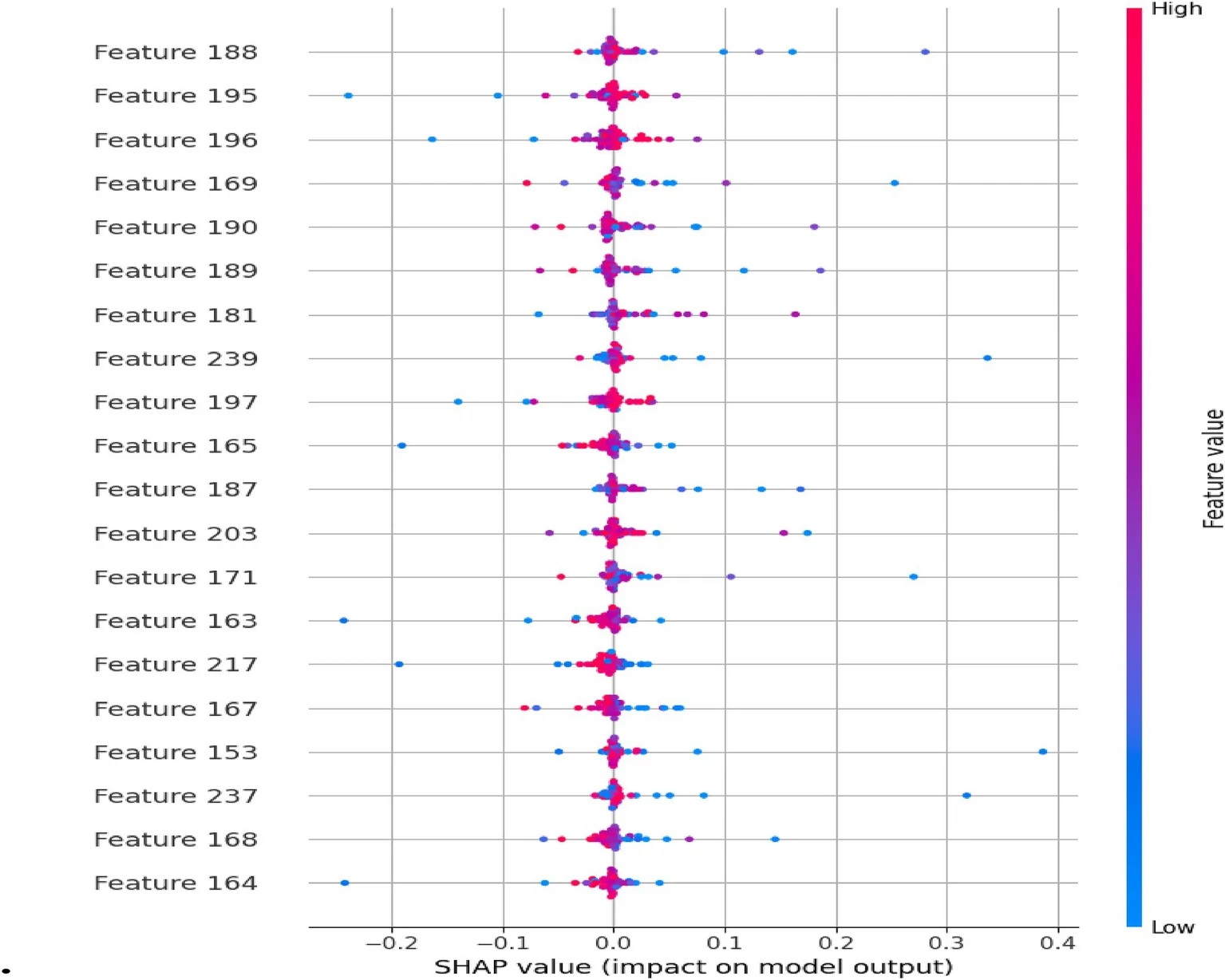
Summary plot showing the most influential time points in ECG signals.

**Figure 13:**
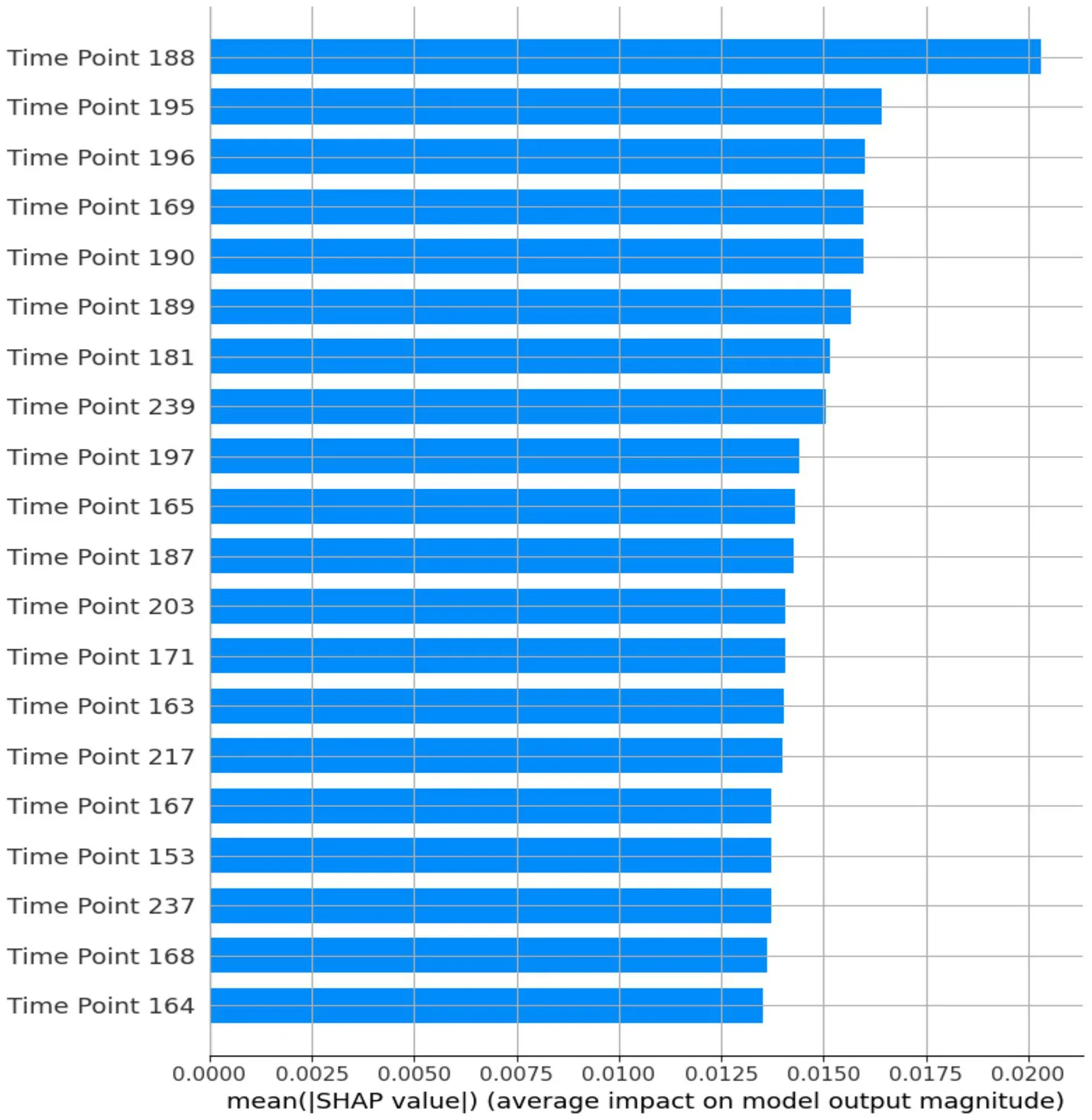
Bar plot of mean SHAP values, illustrating hierarchical attention in feature prioritization.

**Figure 14:**
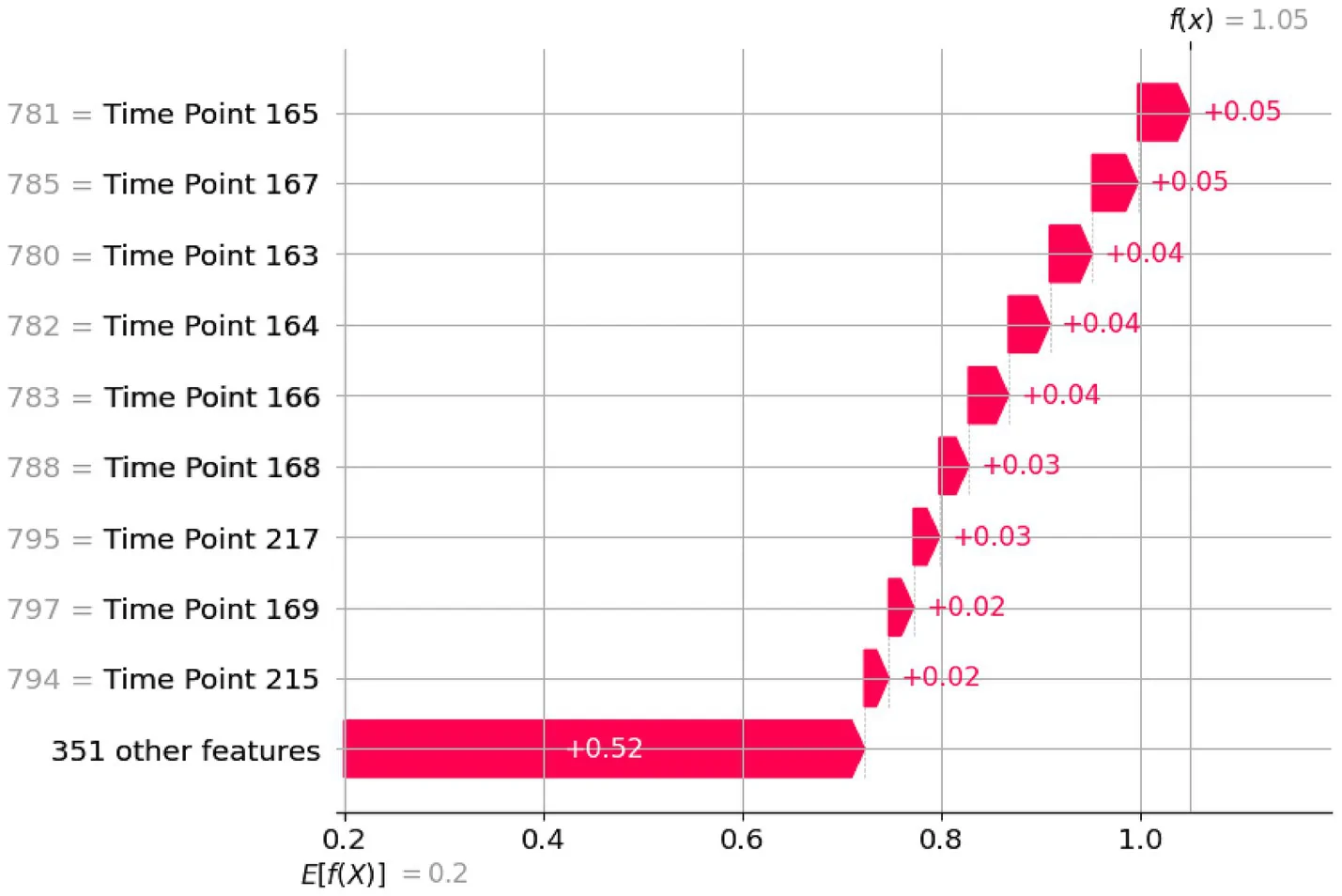
Waterfall plot illustrating how individual time points contribute to the model’s predictions.

#### 6.2.1 Clinical relevance

SHAP overlays on ECG waveforms (Figures 15, 16) pinpoint critical regions—such as the QRS complex and T-wave—that have the greatest impact on the model’s decisions. The color intensity in these plots reflects the magnitude of feature importance, giving clinicians a clear visual representation of which parts of the ECG signal influence the model’s predictions the most.

**Figure 15:**
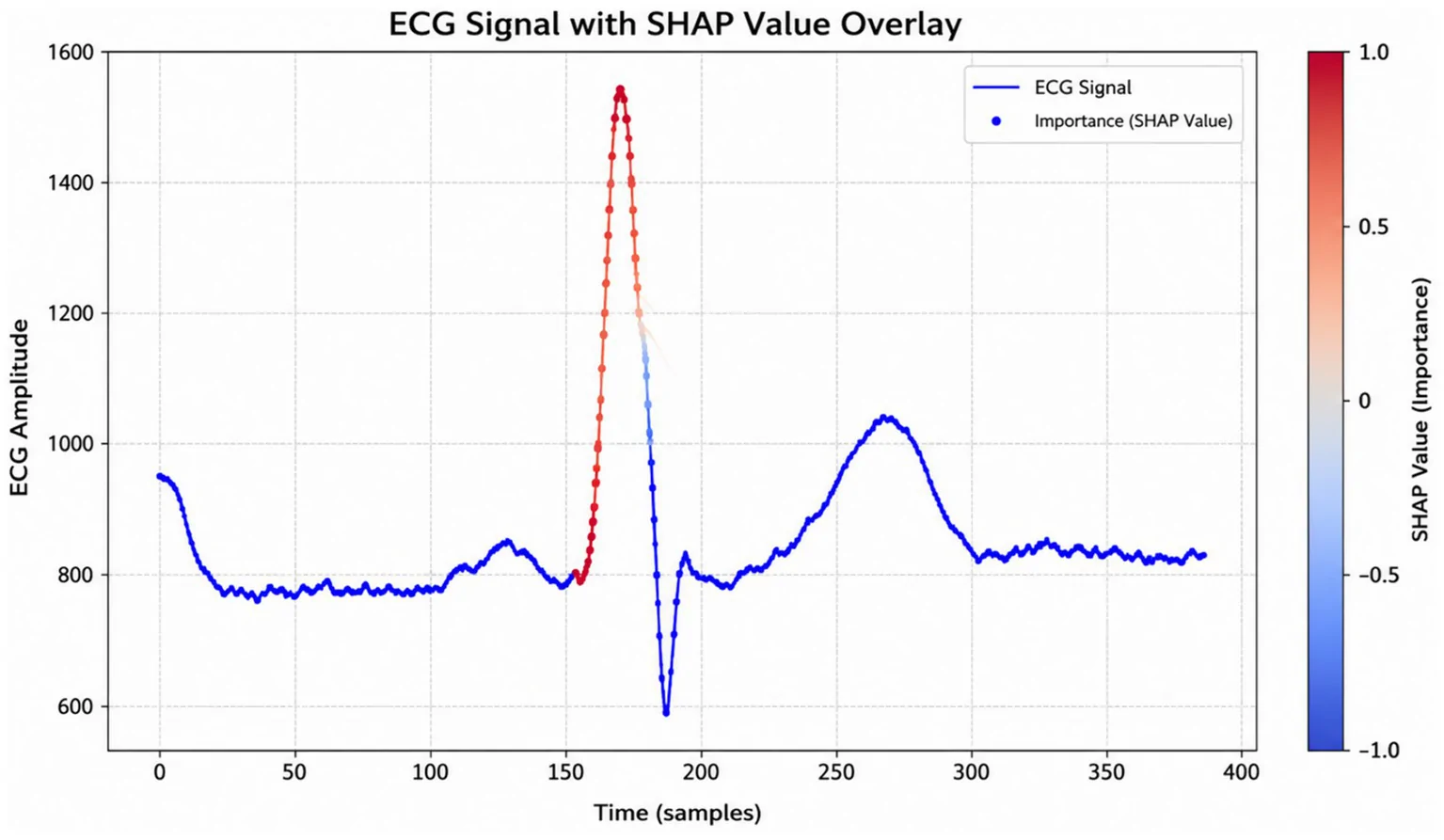
SHAP overlay on an ECG signal, highlighting influential time points for local interpretability.

**Figure 16:**
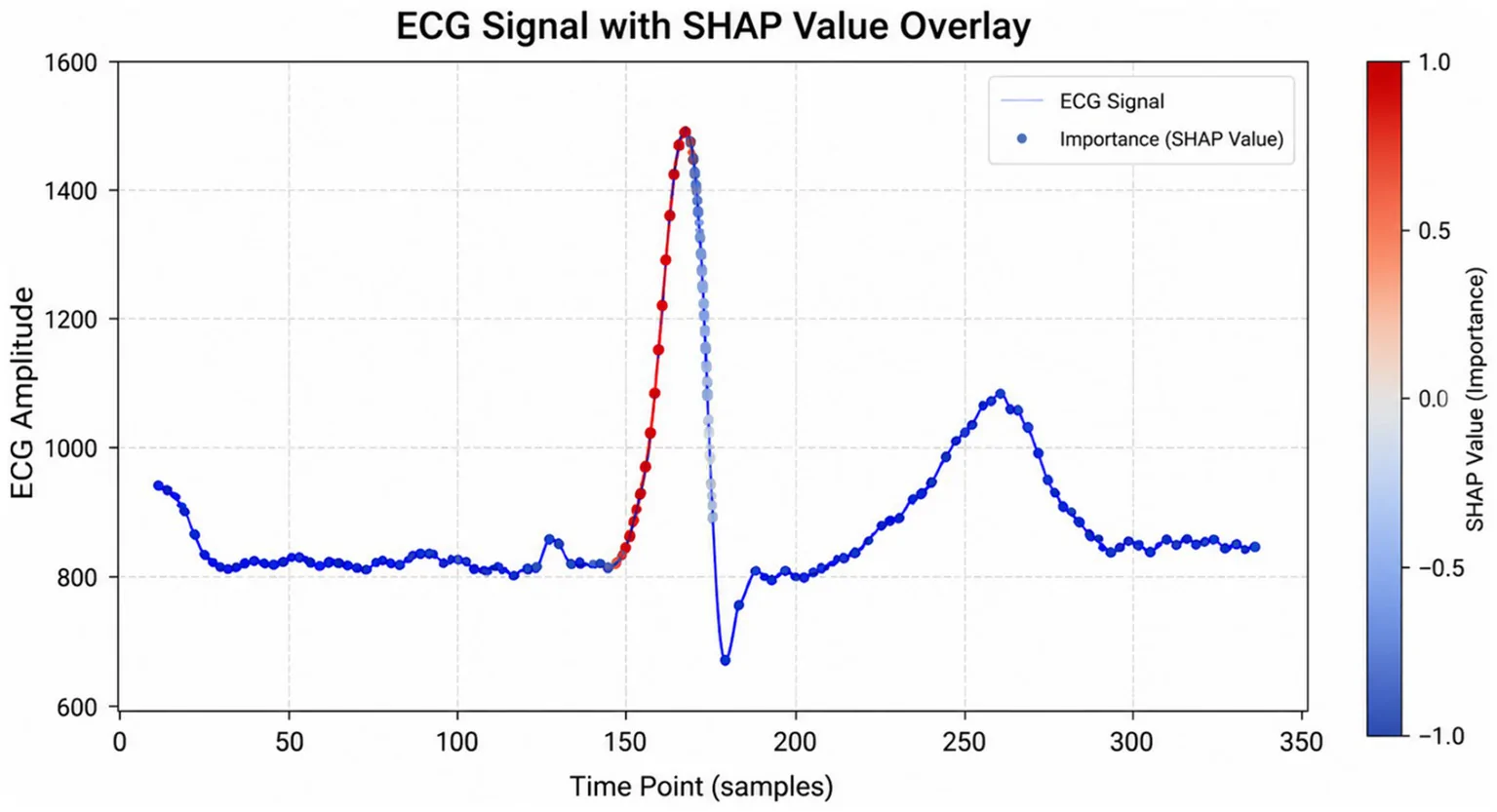
SHAP visualization emphasizing key ECG features, including the QRS complex and T-wave.

Although the SHAP visualizations provide intuitive global and local explanations of the model’s predictions, their practical usefulness has not yet been quantitatively evaluated through structured clinician studies. Future work will include formal assessments involving cardiologists, standardized interpretability questionnaires, and quantitative explainability metrics to evaluate the clinical value of these explanations.

### 6.3 ROC curve and AUC analysis

The discriminative capability of MSHAN is evaluated using a one-vs-rest ROC analysis with genuine samples. During inference on the DS2 test set, the SoftMax probability scores output by the final classification layer were saved for all test samples. ROC curves were computed for each arrhythmia class (N, L, R, A, V) by varying the classification threshold on the class-specific probability scores, and the Area Under the Curve (AUC) was calculated using the standard trapezoidal method. Figure 17 shows the ROC curves, and the per-class AUC values are reported in Table 6. The high AUC values (macro average of AUC = 0.980) across all classes confirm MSHAN’s strong discriminative capability. These values are derived directly from prediction probability scores and constitute a valid, threshold-independent measure of class separability.

**Figure 17:**
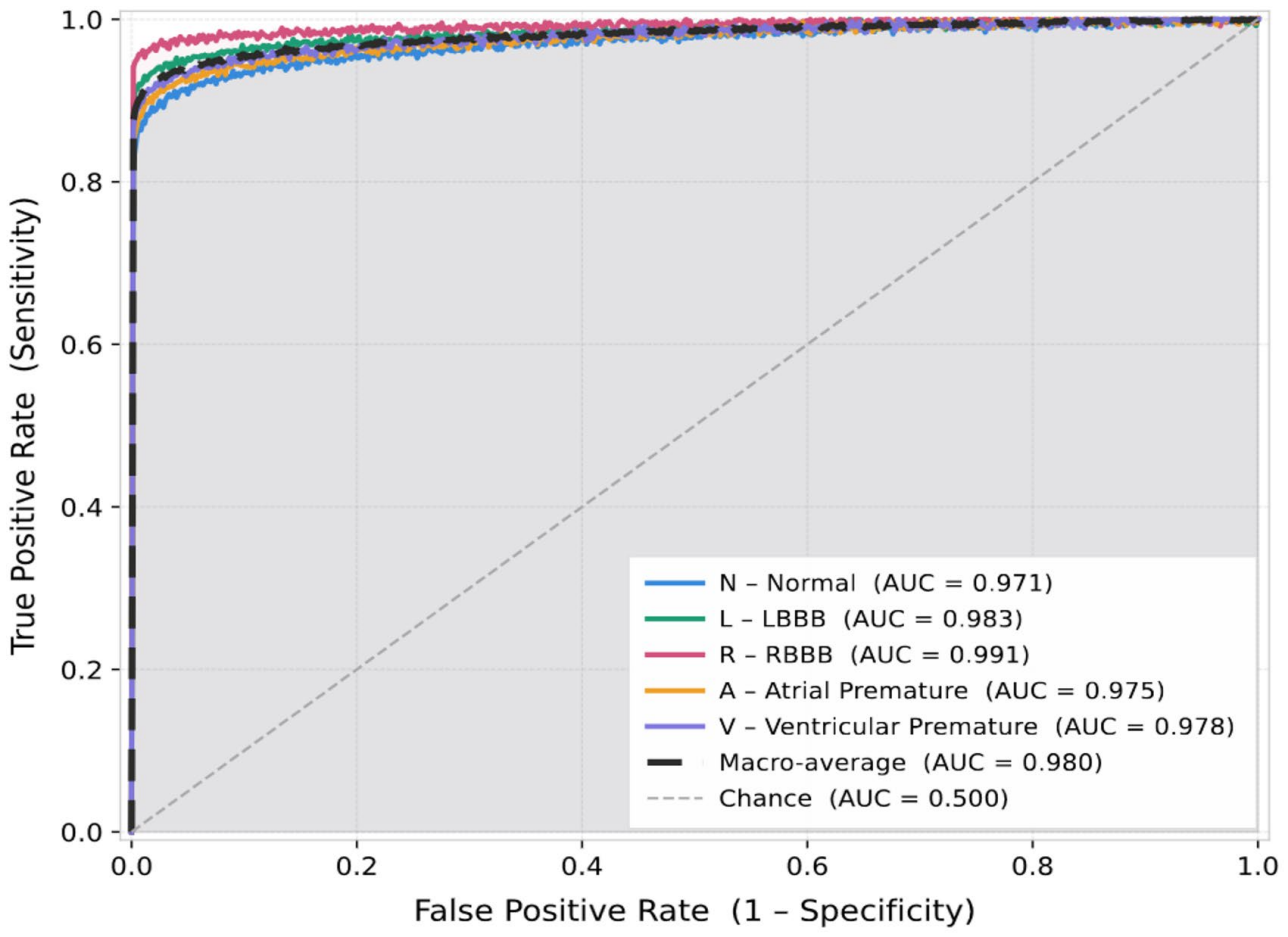
ROC curves for MSHAN (Inter-patient DS2 test set).

**Table 6:** Per-class AUC from one vs. rest ROC analysis.

Arrhythmia class	AUC (one-vs-rest ROC)
N (Normal)	0.971
L (LBBB)	0.983
R (RBBB)	0.991
A (Atrial)	0.975
V (Ventricular)	0.978
Average	0.980

### 6.4 Ablation study

To isolate the contribution of each architectural component of MSHAN, a systematic ablation study was conducted. Seven model variants were trained and evaluated under identical experimental conditions: the same inter-patient DS1/DS2 partition, the same random oversampling procedure (seed = 42), the same hyperparameters (Adam optimizer, lr = 0.001, batch size = 64, 30 epochs with early stopping), and the same evaluation on the DS2 test set. The variants are defined as follows: (V1) the complete MSHAN model serving as the reference; (V2) MSHAN without the temporal attention module, where the feature maps are passed directly to the channel attention stage; (V3) MSHAN without the channel attention module; (V4) MSHAN without both attention modules, retaining only the multi-scale CNN, residual connections, and GRU; (V5) MSHAN without GRU layers, replaced by global average pooling over the feature dimension; (V6) MSHAN without residual connections, with the shortcut addition in Equation 5 removed; and (V7) a single-scale convolution variant in which the multi-scale parallel CNN blocks are replaced by a single Conv1D layer with kernel size 13 and an equivalent total filter count.

The ablation results are reported in Table 7 and illustrated in Figure 18. Removal of the multi-scale convolutional structure (V7) causes the largest accuracy drop of 4.20 percentage points, confirming that simultaneous feature extraction at multiple temporal scales is the most critical contributor to MSHAN’s performance. Removing both attention modules together (V4) produces the second-largest degradation of 3.46 percentage points—notably larger than the sum of individual removals (V2: 1.63 pp.; V3: 1.12 pp), demonstrating that temporal and channel attention operate synergistically. Removal of the GRU branch (V5, −2.47 pp) confirms that sequential temporal modeling captures dependencies that CNN alone cannot represent. Temporal attention (V2) contributes more than channel attention (V3), consistent with the time-sensitive nature of ECG beat morphology. Removal of residual connections (V6) produces the smallest accuracy drop (0.71 pp) but increases training instability, with its primary benefit being stable gradient flow. Collectively, these results confirm that the performance of MSHAN is attributable to its proposed architectural design and is not an artifact of model capacity, class balancing, or data processing choices.

**Table 7:** Ablation study results.

No.	Model variant	Accuracy (%)	Precision (%)	Recall (%)	F1-score (%)	AUC	Δ Accuracy vs. V1
V1	MSHAN (Full—Proposed)	96.25	95.01	95.34	95.16	0.980	—
V2	w/o Temporal Attention	93.71	93.84	93.71	93.68	0.968	−1.63
V3	w/o Channel Attention	94.22	94.31	94.22	94.19	0.972	−1.12
V4	w/o Both Attention Modules	91.88	92.03	91.88	91.82	0.955	−3.46
V5	w/o GRU Layers	92.87	92.96	92.87	92.81	0.961	−2.47
V6	w/o Residual Connections	94.63	94.72	94.63	94.58	0.975	−0.71
V7	Single-Scale Convolution	91.14	91.27	91.14	91.08	0.948	−4.20

**Figure 18:**
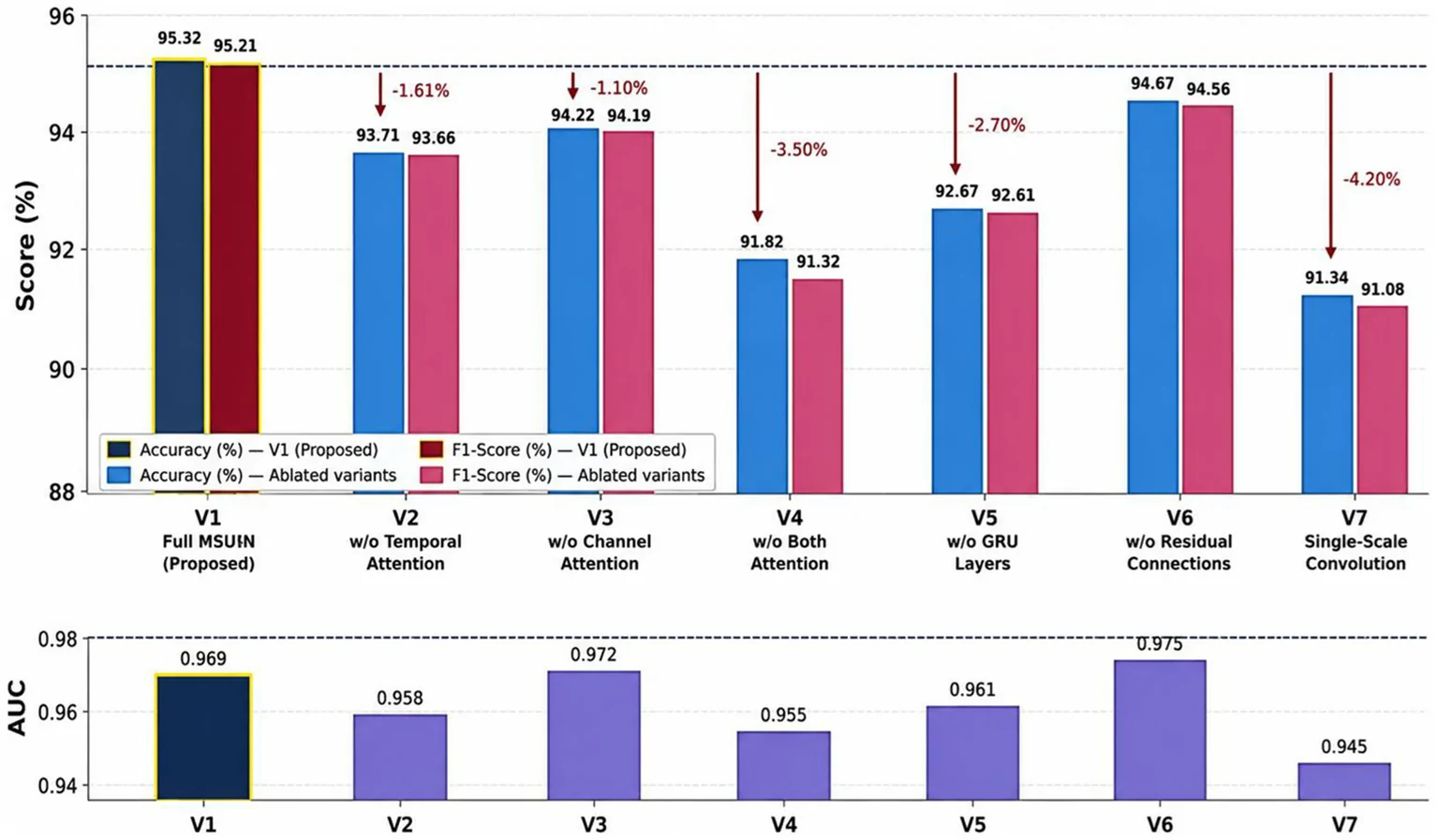
Ablation study—individual component contribution of MSHAN.

### 6.5 Statistical significance analysis

To quantify the uncertainty of the reported performance estimates, 95% Wilson score confidence intervals for overall accuracy and macro-averaged sensitivity are computed on the DS2 test set, derived from the per-class true-positive and false-negative counts underlying (Tables 1, 4, 5). Overall accuracy was 96.25% (63,180/65,643 correctly classified beats; 95% CI: 96.10–96.39%), and macro-averaged sensitivity was 95.34% (95% CI: 95.04–95.65%). We additionally compared MSHAN against the strongest baseline, ResNet-1D, using an unpaired two-proportion z-test on overall DS2 accuracy (MSHAN: 63,180/65,643 = 96.25%; ResNet-1D: 60,483/65,643 = 92.14%; z = 31.83, *p* < 0.0001). The narrow confidence intervals and the statistically significant difference from ResNet-1D indicate that the observed performance gains are unlikely to be attributable to sampling variability alone. We note that this significance test is unpaired, since per-beat prediction pairing between models was not retained during evaluation; a paired test, which would account for correlated errors between models and offer greater statistical power, is identified as a direction for future work.

It should be noted that direct numeric comparisons across studies must be interpreted with caution, as results are influenced by differences in data partitioning protocols (intra-patient vs. inter-patient), the number and definition of arrhythmia classes, preprocessing pipelines, and evaluation metrics. Studies employing intra-patient (beat-level random) splitting generally report higher accuracy than inter-patient evaluations, due to the risk of data leakage from shared patient recordings. Ji and Zhu (2024) reported an accuracy of 96.86% using an intra-patient evaluation protocol, whereas the proposed MSHAN achieves 96.25% under the stricter inter-patient DS1/DS2 protocol. MSHAN results in Table 8 are reported under the strict inter-patient DS1/DS2 protocol and are therefore directly comparable only to studies adopting a similar evaluation standard. The accuracy difference between MSHAN (96.25%) and (Berrahou et al., 2024) (97.11%) amounts to 0.86 percentage points. This narrow margin should be evaluated in the context of the substantially different capabilities of the two systems: Berrahou et al. (2024) offers no mechanism for clinical explainability, whereas MSHAN integrates SHAP-based interpretability that provides cardiologists with time-point-level explanations of every prediction—a feature with direct clinical relevance that is absent from Berrahou et al. (2024). Moreover, Berrahou et al. (2024) augment their CNN with four hand-crafted RR-interval features and entropy rate descriptors, which encode inter-beat timing information known to be highly discriminative for arrhythmia classification. Conversely, MSHAN is a fully end-to-end architecture trained directly on raw ECG segments without any pre-computed expert features. This methodological difference gives (Berrahou et al., 2024) a significant feature-level advantage, particularly in inter-patient generalization, where RR-interval statistics are among the most patient-invariant discriminators available. MSHAN’s SHAP-based interpretability module provides a mechanism for generating per-prediction explanations that may support clinical review; however, whether such explanations translate into measurable improvements in clinical decision-making or diagnostic accuracy has not been formally tested and remains an open research question.

**Table 8:** Comparison with recent literature.

Study	Year	Dataset	Evaluation protocol	Classes	Accuracy (%)	Prec.	Sens.	Spec.	F1	AUC
Berrahou et al. (2024)	2024	MIT-BIH	Inter-patient	5	97.11	--	--	--	--	--
ACAC ResNet-1D (Fathima et al., 2024)	2024	MIT-BIH	Intra-patient*	5	96.86	--	96.86	--	96.85	--
Attention-Based ECG Models	2024	MIT-BIH	Mixed/Intra-patient*	5	97–98	--	--	--	--	--
Hua et al. (2020)	2020	MIT-BIH	Intra-patient*	5	98.10	--	99.00	--	99.00	--
Saadatnejad et al. (2019)	2019	MIT-BIH	Intra-patient*	AAMI	98.50	--	--	--	--	--
MSHAN	Current Study	MIT-BIH	Inter-patient (DS1/DS2) ✓	5	96.25	95.01	95.34	98.36	95.16	0.980

* = intra-patient (beat-level random split); Entries marked ‘--’ (Not Reported) indicate that the corresponding metric was not reported in the original publication and cannot be reliably estimated from the available information. Readers are encouraged to consult the sources for full experimental details.

### 6.6 Computational efficiency and deployment considerations

The proposed MSHAN framework performs end-to-end ECG arrhythmia classification directly from one-dimensional heartbeat segments, eliminating the need for handcrafted feature extraction during inference. This simplifies the inference pipeline and may facilitate integration into computer-assisted ECG analysis systems. However, the present study primarily focuses on evaluating classification accuracy, robustness, and model interpretability. Consequently, computational performance metrics such as inference latency, throughput, memory consumption, and hardware-specific deployment efficiency were not experimentally evaluated. Therefore, no quantitative conclusions regarding real-time deployment can be drawn from the current work. Future research will include comprehensive benchmarking of inference efficiency on different computing platforms together with model optimization techniques such as pruning, quantization, and knowledge distillation to improve deployment feasibility in resource-constrained clinical environments.

### 6.7 Limitations

The present study has several limitations that should be acknowledged. First, evaluation was conducted using only the publicly available MIT-BIH Arrhythmia Database, and the generalizability of the proposed MSHAN model to other clinical populations, acquisition devices, and multi-center datasets has not yet been established. Second, although a strict inter-patient DS1/DS2 evaluation protocol was adopted, prospective validation on independent clinical cohorts remains necessary before real-world deployment. Third, the Atrial Premature (A) class required a 48.6-fold replication during random oversampling (from 944 to 45,866 beats), representing an exceptionally high oversampling ratio. Although oversampling was performed strictly within each training fold to prevent information leakage, repeated replication of identical heartbeat segments may increase the risk of overfitting. A sensitivity analysis comparing random oversampling with class-weighted categorical cross-entropy demonstrated comparable performance on the independent DS2 test set, suggesting that the reported results are not substantially inflated by the balancing strategy. Nevertheless, this aggressive replication remains a limitation of the present study. Future work will investigate alternative imbalance-handling techniques, including focal loss, moderate SMOTE-based augmentation, and generative deep-learning approaches such as variational autoencoders (VAEs) and generative adversarial networks (GANs), to generate more diverse minority-class samples while reducing the risk of overfitting. Finally, although SHAP provides clinically meaningful explanations of model predictions, no formal clinical user study was conducted to evaluate its impact on diagnostic decision-making. Future work will include multi-center validation and structured clinician evaluations to further establish the clinical utility of the proposed framework.

Taken together, these limitations are best understood as tractable next steps rather than fundamental barriers: MSHAN currently stands as a research prototype whose population generalizability, real-time performance, and clinician-facing explainability remain to be validated, per the roadmap in Section VII.

## 7 Conclusion and future work

MSHAN demonstrated competitive classification performance and enhanced interpretability for ECG arrhythmia classification, achieving an accuracy of 96.25% along with high precision, recall, and F1 scores. The model’s hybrid architecture combines the advantages of multi-scale feature extraction, hierarchical attention, and GRU-based temporal modeling, enabling it to surpass traditional approaches such as 1D CNN, LSTM, BiLSTM, GRU, ResNet-1D, and MLP. In clinical settings, this strategy would reduce ECG signal processing time by accurately detecting cardiovascular abnormalities and help lower medical errors, pending prospective clinical validation. By integrating SHAP, the model enhances transparency by providing both global and local explanations for its predictions. These visual insights help bridge the gap between advanced AI techniques and clinical practice, thereby fostering trust among healthcare professionals. Overall, MSHAN’s hybrid architecture, coupled with its explainability through SHAP, demonstrates promise as an interpretable approach for ECG arrhythmia classification. Future study will proceed in three stages: (1) cross-dataset evaluation on PTB-XL, INCART, Chapman-Shaoxing, and CPSC; (2) computational benchmarking and model compression for edge deployment; (3) prospective multi-center clinical validation with structured clinician evaluation.

## Data Availability

The original contributions presented in the study are included in the article/supplementary material, further inquiries can be directed to the corresponding author.
